# Item-specific neural representations during human sleep support long-term memory

**DOI:** 10.1371/journal.pbio.3002399

**Published:** 2023-11-20

**Authors:** Jing Liu, Tao Xia, Danni Chen, Ziqing Yao, Minrui Zhu, James W. Antony, Tatia M. C. Lee, Xiaoqing Hu

**Affiliations:** 1 Department of Applied Social Sciences, The Hong Kong Polytechnic University, Hong Kong, People’s Republic of China; 2 The State Key Laboratory of Brain and Cognitive Sciences, The University of Hong Kong, Hong Kong, People’s Republic of China; 3 Department of Psychology, The University of Hong Kong, Hong Kong, People’s Republic of China; 4 Department of Psychology & Child Development, California Polytechnic State University, San Luis Obispo, California, United States of America; 5 Laboratory of Neuropsychology and Human Neuroscience, Department of Psychology, The University of Hong Kong, Hong Kong, People’s Republic of China; 6 HKU-Shenzhen Institute of Research and Innovation, Shenzhen, People’s Republic of China; Universitat Jaume 1, SPAIN

## Abstract

Understanding how individual memories are reactivated during sleep is essential in theorizing memory consolidation. Here, we employed the targeted memory reactivation (TMR) paradigm to unobtrusively replaying auditory memory cues during human participants’ slow-wave sleep (SWS). Using representational similarity analysis (RSA) on cue-elicited electroencephalogram (EEG), we found temporally segregated and functionally distinct item-specific neural representations: the early post-cue EEG activity (within 0 to 2,000 ms) contained comparable item-specific representations for memory cues and control cues, signifying effective processing of auditory cues. Critically, the later EEG activity (2,500 to 2,960 ms) showed greater item-specific representations for post-sleep remembered items than for forgotten and control cues, indicating memory reprocessing. Moreover, these later item-specific neural representations were supported by concurrently increased spindles, particularly for items that had not been tested prior to sleep. These findings elucidated how external memory cues triggered item-specific neural representations during SWS and how such representations were linked to successful long-term memory. These results will benefit future research aiming to perturb specific memory episodes during sleep.

## Abstract 2

Understanding how individual memories are reactivated during sleep is essential in understanding memory consolidation. This study shows that during human slow-wave sleep, unobtrusively re-presenting memory reminders elicits fine-grained item-specific neural representations that support the formation of long-term memories.

## Introduction

Newly acquired experiences require consolidation to convert initial neural representations into enduring and stable memory [1]. Studies using various techniques (e.g., single-unit recordings, fMRI, electroencephalogram (EEG)) suggest that repeated, covert reactivation of memories during post-learning non-rapid eye movement (NREM) sleep is crucial in memory consolidation [2–8]. Intriguingly, memory reactivation can be manipulated by unobtrusively re-presenting sensory cues associated with wakeful learning during subsequent sleep, a paradigm known as targeted memory reactivation (TMR) [9–12]. TMR allows researchers to determine which memories become reactivated via replaying stimulus-specific memory cues during sleep, thereby bearing promise in modulating declarative, procedural, and emotional memories in both clinical and educational settings [10,13–17]. However, given the challenges in identifying memory-specific neural ensembles during sleep in humans [18], how individual TMR cues reactivate their corresponding memory representations and thereby facilitate long-term memory formation remains far from clear.

Recent studies using multivariate neural decoding methods have advanced our understanding of TMR cue-elicited memory reactivation during sleep. First, cue-elicited neural activity contained task-related or category-level memory representations [19–22]. Second, during NREM sleep, neural representations associated with wakeful retrieval reemerged rhythmically at approximately 1 Hz following TMR cues [23]. However, the extent to which TMR cues can trigger item-level neural representations, a more fine-grained representational format beyond the category-level representations [24], remains elusive. Moreover, although the sleeping brain still shows robust neural responses to external stimuli [25,26], the temporal dynamics and functional significance underlying the processing of memory cues and the reprocessing of cue-target memory during sleep [27] is yet to be established.

Converging evidence suggests that the thalamocortical spindles are instrumental for both endogenous and exogenous memory reactivation during NREM sleep [27,28]. In TMR, enhanced post-cue spindle-related sigma power not only predicted behavioral benefits [29,30] but also was correlated with the distinctiveness of category-level memory representations [20]. Furthermore, the behavioral benefits of TMR are abolished when post-cue sigma power is reduced [31] and when cueing occurs during the spindle refractory period, i.e., approximately 3 to 6 s following a spindle when a spindle is less likely to occur [29]. This evidence prompts an intriguing question of whether spindles in specific time windows can support item-specific memory representations.

An important factor that may modulate the TMR-induced behavioral benefits and spindle-mediated memory consolidation is pre-sleep testing. Given that testing could strengthen memory via retrieval-induced fast consolidation processes [32,33], pre-sleep tested items may be less likely to benefit from the TMR [34]. Indeed, spindles during both spontaneous sleep and sleep TMR preferentially benefited weak rather than strong memories [35–38]. Alternatively, pre-sleep testing may enhance the future relevance and motivational salience of the tested items, which would make these memories preferentially consolidated [39,40]. To reconcile these competing hypotheses, we further examined the relationship between pre-sleep testing and spindle-mediated memory reactivation.

**Fig 1:**
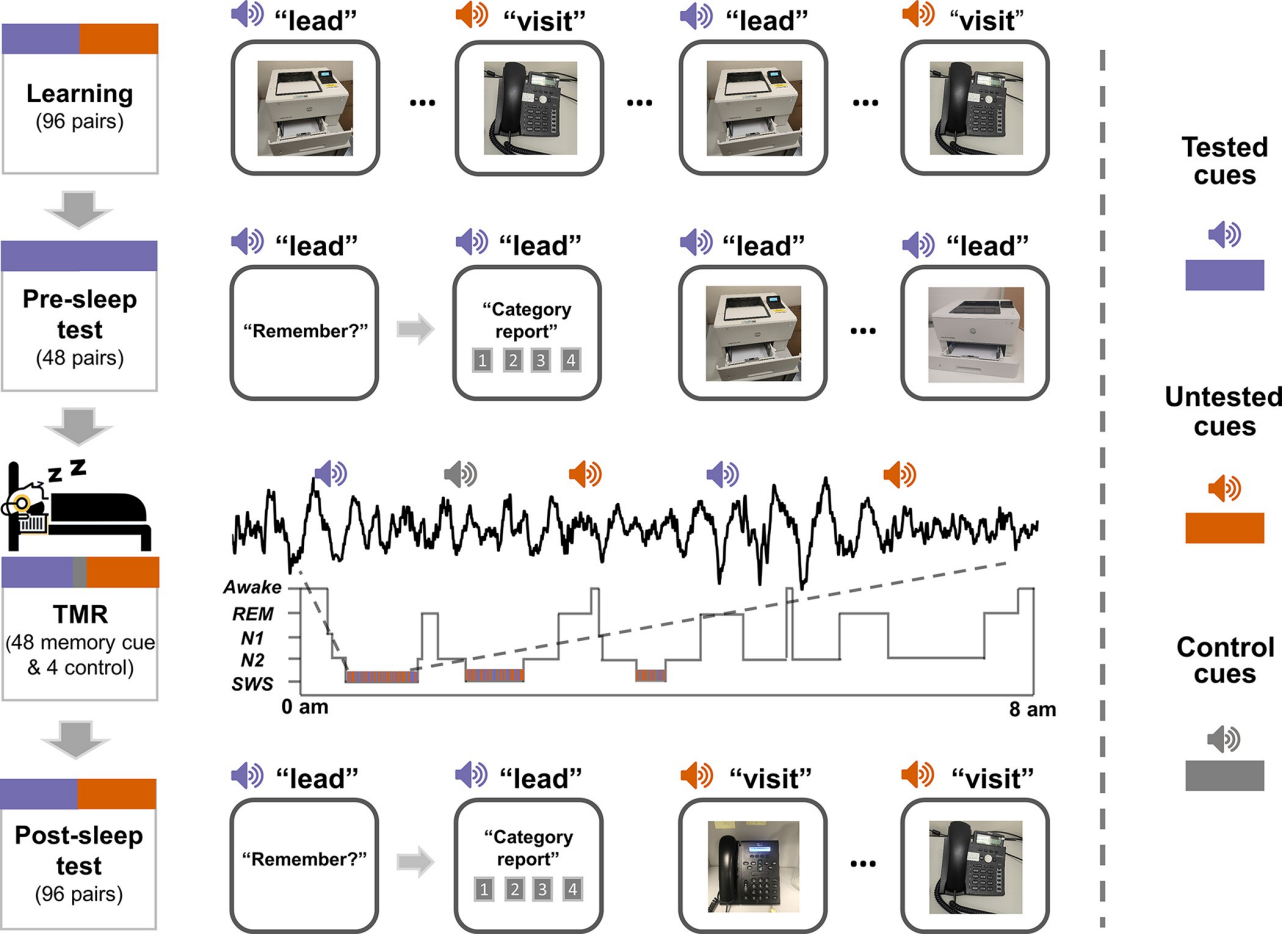
Experimental design. The experiment included 4 phases, i.e., learning, pre-sleep tests (cued recall and cued recognition), TMR during SWS, and post-sleep tests. During the learning phase, participants studied 96 word-picture pairs. During the pre-sleep tests, participants were tested on half of the pairs (tested items) via the cued recall and cued recognition tasks, while the remaining half were not tested (untested items). During TMR, spoken words from half of the tested items (blue speaker icons) and half of the untested items (orange speaker icons), along with 4 control cues (gray speaker icons), were played during the SWS of the overnight sleep. During post-sleep tests on the next morning, memory for all pairs was assessed. SWS, slow-wave sleep; TMR, targeted memory reactivation.

To address these questions, we employed the TMR during post-learning slow-wave sleep (SWS, see Fig 1). Participants (*N* = 30) first studied 96 word-picture (cue-target) pairs, with each pair being repeated 3 times. After a distractor (math) task, participants were tested on half of the materials (i.e., pre-sleep tested items: 48 cue-target pairs) in a cued recall test and a cued recognition test. The remaining 48 cue-target pairs were thus pre-sleep untested items. During subsequent sleep TMR, we replayed 48 auditory memory cues during SWS, which included 24 spoken words from the tested and untested items, respectively. For tested items, pre-sleep cued recall performance was matched for subsequently cued and uncued items (see Materials and methods). For untested items, cues were pseudo-randomly selected. In addition to these 48 memory cues, 4 spoken words that were not paired with any target pictures were included as control cues. During TMR, control cues were played the same number of times as memory cues. On the next morning, participants were tested for all 96 learned word-picture pairs.

Leveraging the representational similarity analysis (RSA), a well-established analytic approach that has been used to examine item-specific neural representations [41–44], we identified 2 functionally distinct item-specific neural representations following memory cues during SWS. Specifically, we found item-specific neural representations (within 0 to 2 s post-cue) for both the memory cues and control cues, yet this early representation bore no relationship with subsequent memory. In contrast, item-specific representations during a later time window (2,500 to 2,960 ms post-cue) predicted subsequent memory: for post-sleep remembered items, cues exhibited greater item-specific neural representations than both the forgotten and control cues. Notably, sleep spindles during this later time window supported the concurrent item-specific neural representations only for pre-sleep untested items, highlighting the intricate role of the spindle in memory reprocessing.

## Results

### Auditory cue-elicited EEG power change during human SWS sleep

**Fig 2:**
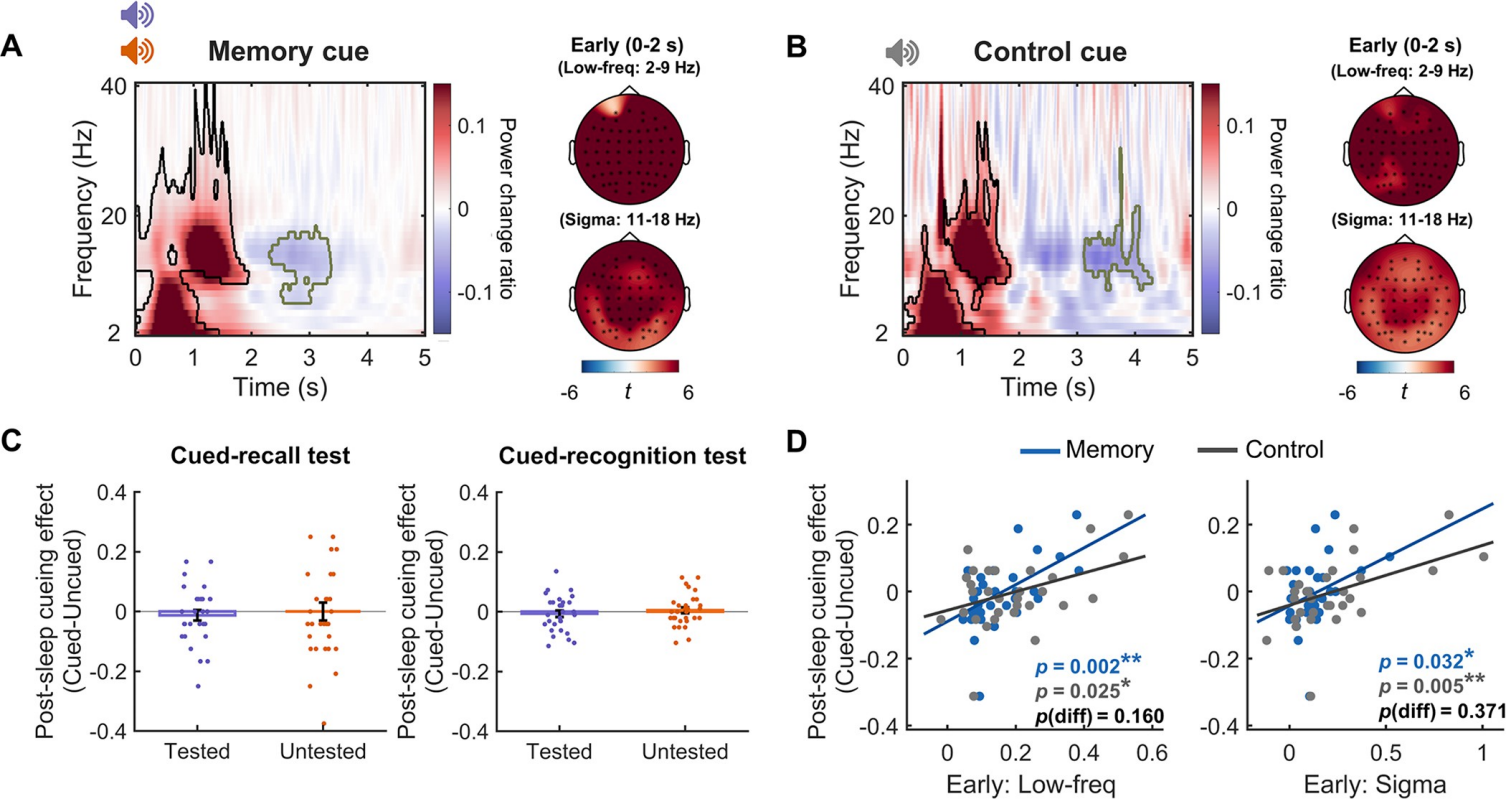
Auditory cue-elicited EEG spectral power changes and their associations with post-sleep cueing effects. **(A)** Left panel: memory cues (for both tested and untested items) enhanced EEG spectral power within an early cluster (circled by the black line), followed by decreased sigma power in a late cluster (circled by the green line), compared to the pre-cue baseline (500–1,000 ms prior to cue onset). Right panel: topographical *t*-value plots for the memory cue-elicited low-frequency range and sigma band power change in the early cluster. **(B)** Left panel: control cues enhanced EEG spectral power within the 2 early clusters (circled by the black line), followed by decreased sigma power in a late cluster (circled by the green line), compared to the pre-cue baseline. Right panel: topographical *t*-value plots for the control cue-elicited low-frequency range and sigma band power change in the early clusters. **(C)** No significant post-sleep cueing effect for pre-sleep tested and untested items in either the post-sleep cued recall task or cued recognition task. **(D)** Both memory cue and control cue-elicited EEG power in the low-frequency range and the sigma band in the early cluster positively predicted post-sleep cueing effects (all cued—uncued items, including both tested and untested items) in the cued recall test. The prediction effect did not differ between memory and control cues. The *p*-values indicate the significance of robust linear regression that minimized the potential influence of statistical outliers. *: *p* < 0.05; **: *p* < 0.01; ***: *p* < 0.001. The data underlying this figure can be found in S1 Data. EEG, electroencephalogram.

We played 52 unique auditory cue words during SWS, with 48 memory cues (24 cues from pre-sleep tested pairs and 24 cues from pre-sleep untested pairs) and 4 control cues that were familiarized but not paired with any target pictures in the pre-sleep learning phase. On average, each cue was presented 8.92 (SD: 3.24) times (see Materials and methods). To confirm that the auditory cues were indeed processed during sleep, we first examined the cue-elicited EEG spectral power change. The result revealed that memory cues, including both the tested and untested cues, significantly enhanced 2 to 40 Hz EEG power during the first 1,960 ms as compared to the pre-cue baseline (i.e., 500 to 1,000 ms prior to cue onset) (*p*_cluster_ < 0.001, see Fig 2A, corrected for multiple comparisons using a cluster-based permutation test, see Materials and methods). Notably, 2 prominent frequency ranges emerged: a low-frequency range of 2 to 9 Hz and an extended sigma band of 11 to 18 Hz. Following this early cluster, we observed a significant reduction of sigma power in a late cluster (2,320 to 3,380 ms post-cue *p*_cluster_ = 0.005), which potentially reflected the spindle refractoriness [29]. Further analyses showed that pre-sleep testing did not modulate cue-elicited EEG power on the above clusters (all *p*s_FWER_ > 0.700, corrected for post hoc comparisons using family-wise error rate, FWER, see S1 Fig).

We next examined whether memory cue-elicited EEG power changes were associated with post-sleep cue-target memory performance. We adopted a subsequent memory approach: Within each participant, we categorized cues based on whether their associated targets were successfully recalled or not during the post-sleep cued recall test (remember versus forget). We next contrasted cue-elicited EEG power elicited by cues between post-sleep remembered versus forgotten items within the identified early (0 to 1,960 ms) and late clusters (2,320 to 3,380 ms, see Fig 2A), respectively. The results revealed no significant EEG power differences between auditory cues of post-sleep remembered versus forgotten items (all *p*s_FWER_ > 0.107, see S1 Fig).

Examining the EEG power locked to the control cues similarly showed enhanced EEG power in the low-frequency range and the sigma band within the first 2 s (both *p*s_cluster_ < 0.001), followed by sigma power reduction (3,130 to 4,300 ms, *p*_cluster_ = 0.006) (Fig 2B). Importantly, directly comparing cue-elicited EEG power between memory cues and control cues revealed no significant differences (*p*_cluster_ > 0.441, S1 Fig). Note that we obtained similar results after matching trial numbers between memory cues and control cues (S1 Fig). These results suggested that the univariate EEG power changes reflect how the sleeping brain responds to the auditory cues, regardless of memory cues or control cues.

Having shown that the sleeping brain did respond to external auditory cues, we next examined whether cueing improved memory performance as compared to the uncued items. Here, we primarily focused on cued recall performance (see Materials and methods), given its sensitivity to TMR manipulations [10]. Given that the pre-sleep recall performance was balanced between cued versus uncued items, we examined the cueing effect based on the post-sleep recall performance. A 2 (tested versus untested) by 2 (cued versus uncued) repeated measures ANOVA revealed a significant main effect of pre-sleep testing (*F*(1,29) = 186.97, *p* < 0.001), but neither a significant main effect of cueing (*F*(1,29) = 0.11, *p* = 0.739, Fig 2C) nor the interaction effect (*F*(1,29) = 0.15, *p* = 0.700). For post-sleep recognition, the same analysis yielded no significant effects (all *p*s > 0.092, Fig 2C, see also S2 Fig). Despite the absence of significant cueing effects at the group level, notable individual differences were observed in the cued recall task (Fig 2C). Robust linear regression analysis revealed that across participants, both the memory cue and the control cue-elicited early EEG power (within the first 2 s post-cue) but not the later sigma power positively predicted the post-sleep cueing effects (early low-frequency [2 to 9 Hz]: adjusted *R*^2^ > 0.13, all *p*s < 0.026; early sigma [11 to 18 Hz]: adjusted *R*^2^ > 0.12, all *p*s < 0.032, Fig 2D; late sigma: adjusted *R*^2^ < 0.064, all *p*s > 0.095). Directly comparing the prediction effects of memory cues and control cues showed no significant differences (all *p*s > 0.159). To investigate whether pre-sleep testing modulated the relationship between cue-elicited early EEG and post-sleep cueing effect, we repeated the same analyses for pre-sleep tested items and untested items, respectively. The results showed that individual differences in both the memory cue and control cue-elicited early EEG power significantly predicted post-sleep cueing effects for pre-sleep untested items (all *p*s < 0.023), but not for tested items (all *p*s > 0.470, S3 Fig). Moreover, this prediction effect was significantly higher for untested items than for tested items (both *p*s < 0.026).

Together, these results suggested that auditory cues, regardless of memory cues or control cues, elicited an increased early EEG power (within the first 2 s post-cue), followed by a decreased sigma power. The observed changes in EEG power were not associated with the post-sleep memory performance of individual cue-target pairs within each participant. Importantly, across participants, individuals with stronger early EEG responses to cues were more likely to show larger post-sleep TMR cueing effects, especially for pre-sleep untested items.

### Item-specific representations for both memory cues and control cues in an early time window

**Fig 3:**
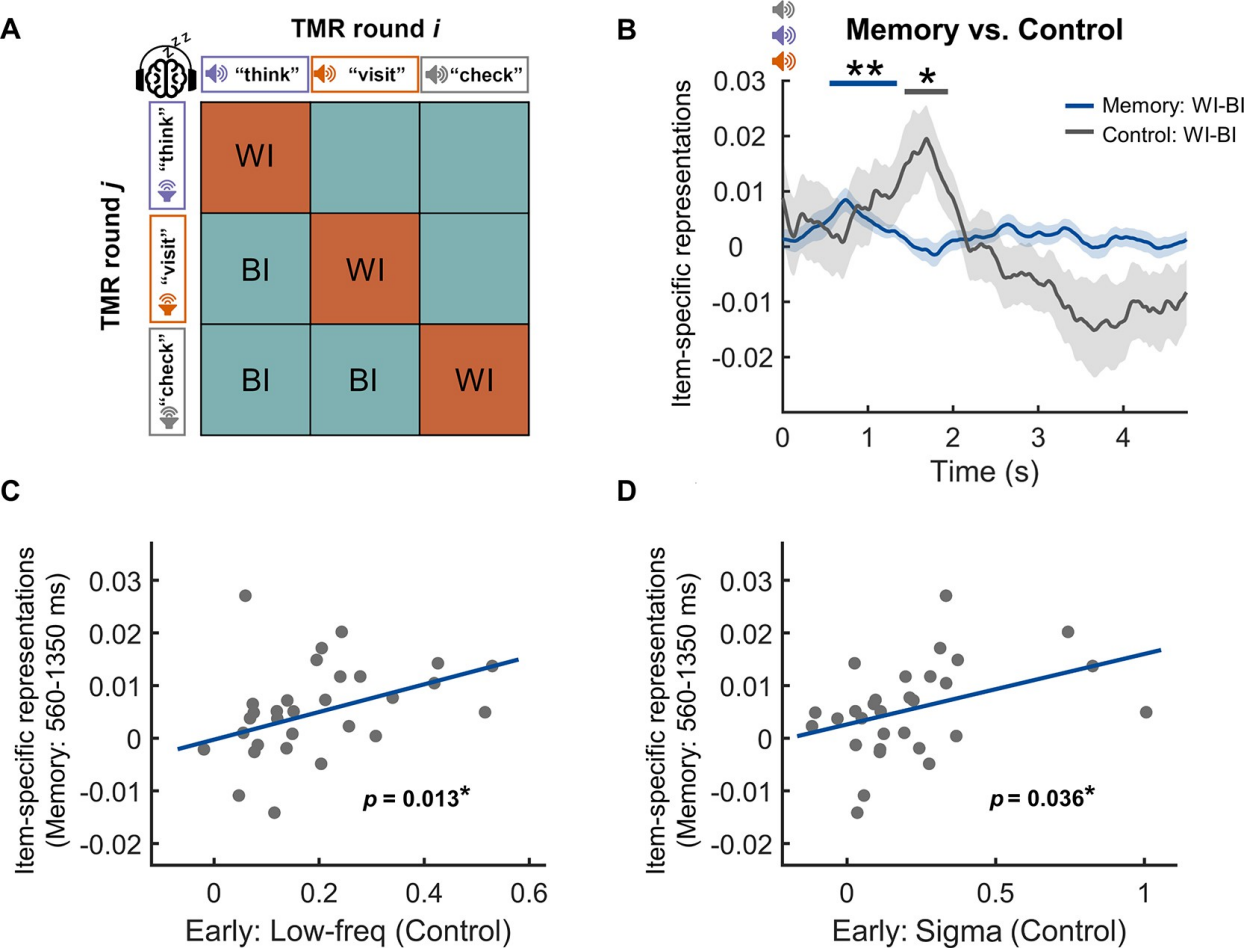
Item-specific representations following TMR cues during SWS. **(A)** RSA scheme across different TMR blocks. Artifact-free raw EEG data pattern was correlated between trials with the same auditory cues (WI similarity) and between trials with different auditory cues (BI similarity). Item-specific representations were examined by contrasting the WI similarity versus BI similarity. **(B)** Item-specific representations for memory cues and control cues were significant in the 560–1,350 ms (blue horizontal bar on the top) and 1,440–1,950 ms (gray horizontal bar on the top) time windows after cue onset, respectively. **(C, D)** Control cue-elicited low-frequency EEG power and sigma band EEG power in the early cluster (<2 s, see Fig 2B) predicted the item-specific representations for memory cues in the 560–1,350 ms time window, respectively. *: *p* < 0.05; **: *p* < 0.01. The data underlying this figure can be found in S1 Data. BI, between-item; EEG, electroencephalogram; RSA, representational similarity analysis; SWS, slow-wave sleep; TMR, targeted memory reactivation; WI, within-item.

We next examined whether cue-elicited EEG would contain item-specific representations. To test this hypothesis, we employed the multivariate RSA to examine the neural pattern similarity between trials of the same cues (within-item [WI] similarity) as well as the similarity between trials of different cues (between-item [BI] similarity) (Fig 3A) [42,45]. Item-specific neural representations were indicated by significantly greater WI similarity than BI similarity. Specifically, we computed the representational similarity values between trials by correlating the cue-elicited raw EEG pattern (filtered between 0.5 and 40 Hz) across all channels. This analysis was conducted within consecutive time windows of 500 ms, with a sliding step of 10 ms, during a 5-s post-cue period. The WI and BI similarities were calculated between trials from 2 different TMR blocks to control for the temporal proximity effect. Our analysis revealed significant item-specific neural representations (i.e., WI > BI similarities) in a 560 to 1,350 ms time window post-cue (*p*_cluster_ = 0.008, Fig 3B). However, averaged item-specific representations within this cluster did not differ between post-sleep remembered and forgotten items (*t*(29) = 1.65, *p* = 0.110), suggesting that the early item-specific representations did not contribute to post-sleep memory.

The RSA on control cue-elicited EEG activity similarly revealed significant item-specific neural representations during 1,440 to 1,950 ms after cue onset (*p*_cluster_ = 0.040, Fig 3B). Moreover, a repeated measures ANOVA on item type (memory versus control) and item-specific representations (WI versus BI) during whole post-cue time windows did not reveal any significant clusters (*p*_cluster_ > 0.085). Notably, these results remained consistent after matching trial pair numbers between WI and BI conditions and between memory and control cues (S4 Fig). The relatively delayed onset of item-specific representations for control cues may reflect the cost of processing speed given that the control cues were less studied pre-sleep than memory cues [46].

These results suggested that the early item-specific representations were mainly associated with auditory cue processing. To further confirm this possibility, we performed a linear regression analysis to examine whether control cue-elicited early EEG power change (i.e., within the first 2 s post-cue, Fig 2B) could predict memory cue-elicited early item-specific representations (i.e., 560 to 1,350 ms post-cue, Fig 3B). The results revealed that the control cue-elicited early EEG power in both the low-frequency range and the sigma band positively predicted the memory cue-elicited early item-specific representations (low-frequency: adjusted *R*^2^ = 0.18, *p* = 0.013; sigma: adjusted *R*^2^ = 0.12, *p* = 0.036; see Fig 3C and 3D).

Together, these results provided converging evidence that the early item-specific neural representations (within the first 2 s) predominantly reflected effective processing of individual auditory cues, given that these early representations were not associated with post-sleep memory, and that they were not different between memory cues and control cues.

### Greater item-specific representations for post-sleep remembered items than forgotten items in a late time window

**Fig 4:**
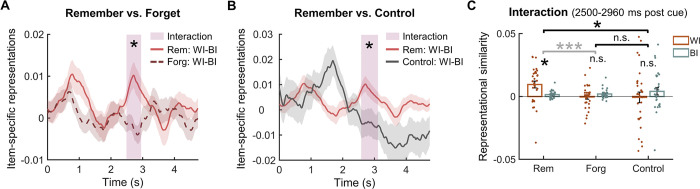
Item-specific representations in a later time window were associated with post-sleep memory performance. **(A)** Item-specific representations following cues of post-sleep remembered items were greater than that following cues of post-sleep forgotten items in a 2,500–2,960 ms time window (shaded rectangle). **(B)** Item-specific representations following cues of post-sleep remembered items were greater than that following control cues in a highly overlapped late time window (2,590–3,120 ms). **(C)** Within the shaded cluster in A (i.e., 2,500–2,960 ms), WI similarity was significantly greater than the BI similarity for cues of remembered items, while there were no differences between WI and BI similarity for cues of forgotten items or control cues. Item-specific representations for cues of remembered items were greater than both the item-specific representations for cues of forgotten items and for control cues, while the item-specific representations for cues of forgotten items were not significantly different from the control cues. *: *p* < 0.05; ***: *p* < 0.001; n.s.: not significant. The data underlying this figure can be found in S1 Data. BI, between-item; WI, within-item.

Having established that EEG elicited by memory cues contained item-specific representations, we next examined our key question: whether memory cues elicited item-specific representations that contribute to the successful post-sleep cue-target memory. We compared the item-specific representations (i.e., WI minus BI similarity) between memory cues of post-sleep remembered items and memory cues of forgotten items. The results revealed a significant positive cluster (2,500 to 2,960 ms after cue onset), such that memory cueing during SWS elicited greater item-specific representations for post-sleep remembered items than forgotten items (*p*_cluster_ = 0.025, Fig 4A). Again, results remained consistent after we matched the trial pair numbers across RSA conditions (i.e., WI remember, BI remember, WI forget, and BI forget; see S4C Fig). Notably, the observed item-specific representations were above and beyond category-level representations, given that the WI similarity was greater than the BI similarity even when the BI items were drawn from within-category (WC) trial pairs (i.e., WC similarity, see S5 Fig).

We next explored which EEG frequency band may drive the item-specific representations. We repeated the item-specific representation analysis using the EEG filtered in different frequency bands (i.e., slow oscillation (SO), slow-wave activity, theta, alpha, sigma, and gamma). We found that both early (560 to 1,350 ms) and late (2,500 to 2,960 ms) item-specific representations following memory cues were mainly contributed by the 0.5 to 4 Hz slow-wave activity (for details, see S6 Fig).

To further confirm that the late (2,500 to 2,960 ms post-cue) item-specific representations were related to cue-target memory reprocessing, we compared item-specific representations for post-sleep remembered memory cues with control cues. The results again revealed that post-sleep remembered items showed significantly greater item-specific representations than control items in a highly overlapped time window (2,590 to 3,120 ms post-cue, *p*_cluster_ = 0.044, see Fig 4B). We next systematically examined the item-specific representation for memory cues of post-sleep remembered/forgotten items and for control cues within the 2,500 to 2,960 ms late time window. We found that WI similarity was significantly greater than BI similarity for cues of remembered items (*t*(29) = 3.01, *p*_FWER_ = 0.016), while there was no significant difference between WI and BI similarity for cues of forgotten items (*t*(29) = −1.28, *p*_FWER_ = 0.637) or for control cues (*t*(29) = −0.95, *p*_FWER_ = 1.000) (Fig 4C). Furthermore, item-specific representations for cues of remembered items were greater than that for control cues (*F*(1,29) = 6.31, *p* = 0.018), while there was no significant difference between item-specific representations for cues of forgotten items and control cues (*F*(1,29) = 0.20, *p* = 0.657). In addition, we found that pre-sleep testing did not modulate the item-specific representations (*p*_cluster_ > 0.383, see S7 Fig). Taken together, these results suggested that the late (2,500 to 2,960 ms post-cue) item-specific representations were associated with successful long-term associative memory.

### Spindle activity preferentially supports the late item-specific representations for pre-sleep untested items

**Fig 5:**
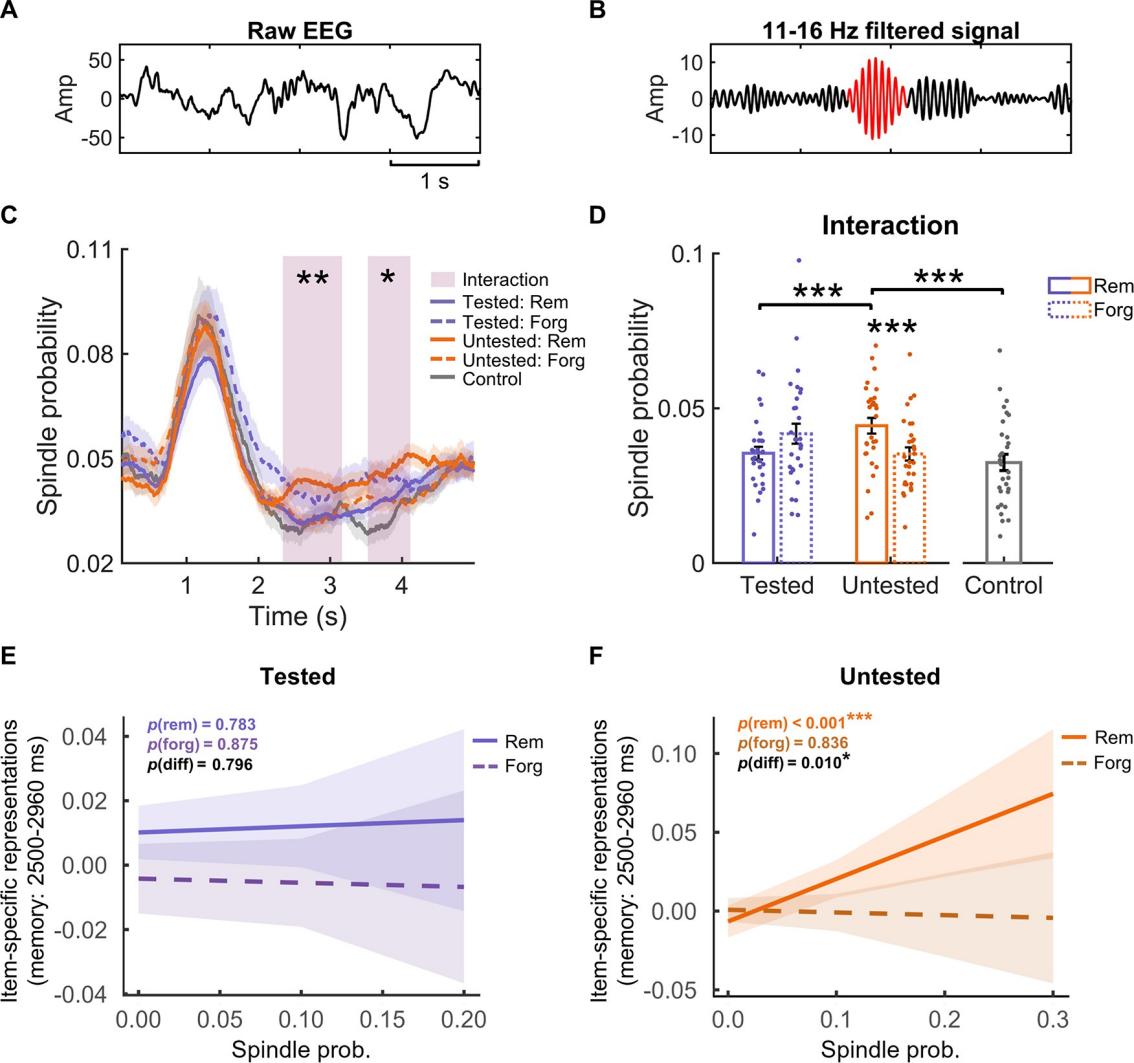
The relationship between spindles and item-specific representations in the overlapped late time window (i.e., 2,500–2,960 ms post-cue). **(A)** An example of raw EEG data used for spindle detection. **(B)** EEG signals in (A) filtered between 11 and 16 Hz, with a spindle event highlighted in red. **(C)** Significant interaction effects between pre-sleep testing (tested vs. untested) and subsequent memory effect (remembered vs. forgotten items) on spindle probability in 2 clusters (2,344–3,166 ms and 3,526–4,118 ms, highlighted in shaded rectangles) following TMR cues. **(D)** Post hoc analyses were performed on the averaged spindle probabilities across the 2 interaction clusters (shaded rectangle in C). For untested items, post-sleep remembered items elicited greater spindle probability than forgotten items. Moreover, the spindle probability for post-sleep remembered untested items was greater than that for both the post-sleep remembered tested items and control items. **(E)** Among tested items, the spindle probability did not predict the item-specific representations for either post-sleep remembered or forgotten items. **(F)** Among untested items, the spindle probability significantly predicted the item-specific representations for post-sleep remembered items but not for forgotten items, with the effect for post-sleep remembered items being greater than that for forgotten items. *: *p* < 0.05; **: *p* < 0.01; ***: *p* < 0.001. The data underlying this figure can be found in S1 Data. EEG, electroencephalogram; TMR, targeted memory reactivation.

Previous TMR studies suggest that memory reprocessing during sleep is tightly linked with post-cue spindle activity [20,27,29]. After identifying memory-related item-specific representations, we next examined how spindle activity is related to such item-specific representations and whether pre-sleep testing could affect their relationship. To address this question, we first detected discrete spindles on individual trials (Fig 5A and 5B, see Materials and methods) and then calculated spindle probability across trials for each time point. We then conducted pre-sleep testing (tested versus untested) by post-sleep memory (remember versus forget) repeated measures ANOVA on spindle probabilities and identified 2 relatively late clusters showing significant interactions (first cluster: 2,344 to 3,166 ms, *p*_cluster_ = 0.002; second cluster: 3,526 to 4,118 ms, *p*_cluster_ = 0.026; Fig 5C). Averaging spindle probability across these 2 clusters, we found that remembered items elicited higher spindle probability than forgotten items only among untested items (*t*(29) = 4.50, *p*_FWER_ < 0.001), but not among tested items (*t*(29) = −2.50, *p*_FWER_ = 0.147, Fig 5D). Moreover, untested remembered items elicited higher spindle probability than both tested remembered items (*t*(29) = 4.51, *p*_FWER_ < 0.001) and control items (*t*(29) = 5.69, *p*_FWER_ < 0.001, Fig 5D), while no significant difference was found between the latter 2 conditions (*t*(29) = 1.42, *p*_FWER_ = 1.000). To ensure that the observed spindle activity differences in the late clusters were not due to the leakage of early spindles (e.g., longer duration or delayed onset of earlier spindles), we conducted control analyses on spindles from the early time window (0 to 2 s) and found no significant interactions for either the duration or the onset of early spindles (all *p*s > 0.370, see S8 Fig). In addition, spindles during an extended time window including these 2 clusters (i.e., 2,200 to 4,200 ms) were significantly coupled to the up-state of SOs for all tested and untested items (Rayleigh tests, all *p*s < 0.045, see S9 Fig).

We next examined the relationship between spindles and the cue-target memory-related late item-specific representations. Notably, increased spindle probabilities (2,344 to 3,166 ms and 3,526 to 4,118 ms in Fig 5C) tended to co-occur with the late item-specific representations (2,500 to 2,960 ms in Fig 4A). We then employed a linear mixed-effect model to examine how spindles were related to item-specific representations in the overlapping time window (i.e., 2,500 to 2,960 ms) for individual tested and untested items, respectively. We found that among untested items, cue-elicited spindle probability positively predicted item-specific representations for post-sleep remembered items (*β* = 0.26, *t*(239) = 3.678, *p* < 0.001, Fig 5F) but not for forgotten items (*β* = −0.02, *t*(472) = −0.21, *p* = 0.836). Moreover, this effect was significantly greater for post-sleep remembered than for forgotten items (*β* = 0.29, *t*(716) = 2.57, *p* = 0.010). In contrast, no significant effects were found among tested items (remember: *β* = 0.02, *t*(432) = 0.28, *p* = 0.783; forget: *β* = −0.01, *t*(258) = −0.16, *p* = 0.875, Fig 5E). These results suggested that sleep spindles preferentially supported the late, memory-related item-specific representations for untested items.

## Discussion

**Fig 6:**
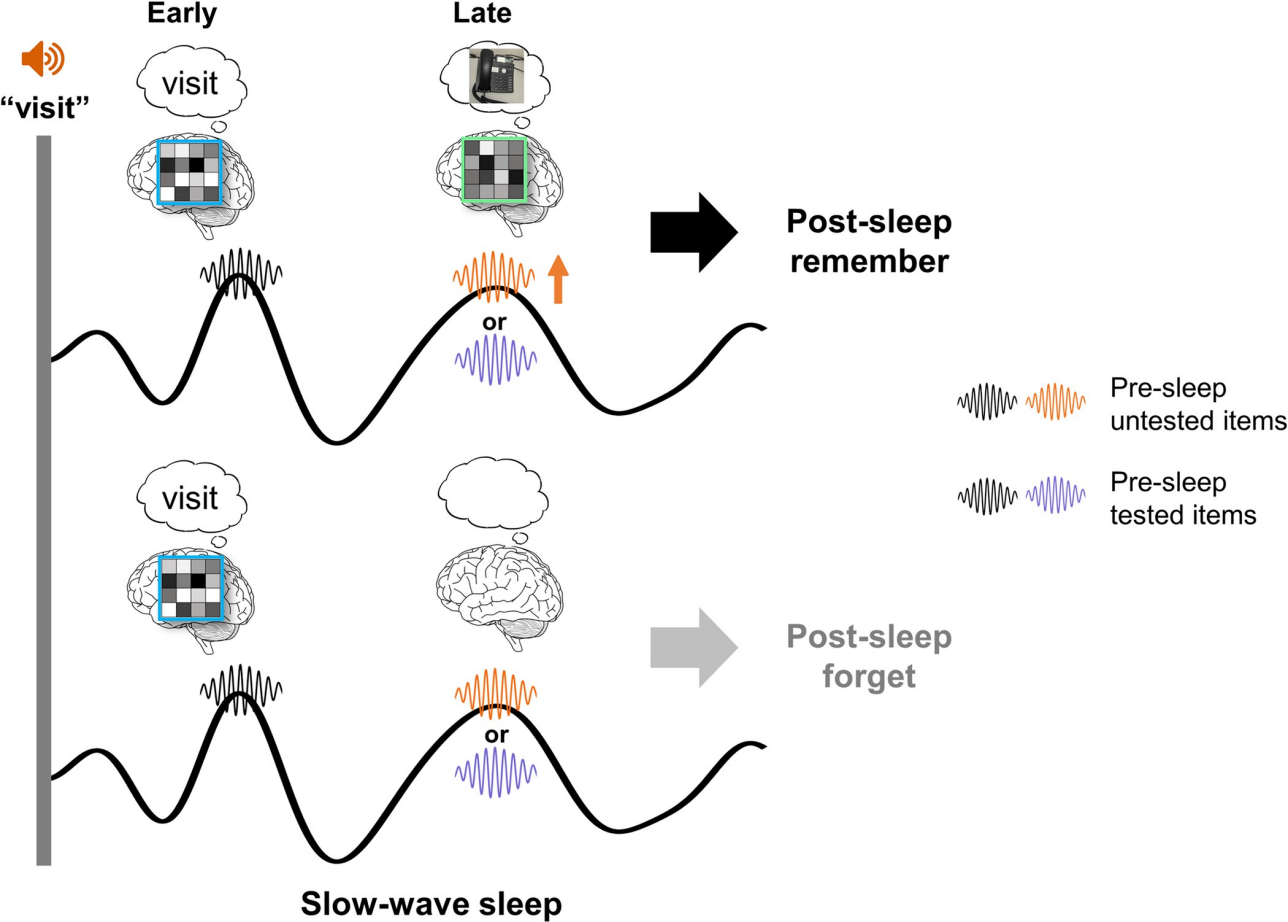
A schematic depiction of item-specific neural representations following TMR cues that support long-term memory. During SWS, sensory memory cues elicit early item-specific neural representations regardless of subsequent memory performance, indicating the processing of sensory cues. Critically, item-specific neural representations in a later time window are associated with successful post-sleep memory performance, indicating memory reprocessing. Furthermore, enhanced spindle activity coupled to the up-states of SOs in a later time window supports the concurrent item-specific representations for pre-sleep untested items. SO, slow oscillation; SWS, slow-wave sleep; TMR, targeted memory reactivation.

We asked fundamental yet unanswered questions in cue-triggered, exogenous memory reactivation: how individual memory cues reactivate the corresponding memory representations in the sleeping human brain, thereby supporting long-term memory. Using TMR and multivariate representational similarity analyses (RSA), we found fine-grained item-specific representations following TMR cues. These results extended previous sleep and TMR studies employing the multivariate decoding method or RSA to identify category-level representation [4,5,20,21] or lateralized features of left versus right motor imagery [22,47]. More critically, we delineated how memory cues elicited temporally segregated and functionally distinct item-level neural representations. Specifically, both memory cues and control cues elicited early item-specific neural representations (within the first 2 s post-cue), likely reflecting the processing of auditory cues. Notably, in a late time window (2,500 to 2,960 ms post-cue), item-specific representations were associated with post-sleep successful recall of cue-target pairs, indicating cue-target memory reprocessing. Critically, increased spindles during this late time window preferentially supported concurrent item-specific representations yet only among pre-sleep untested items, highlighting that pre-sleep testing can modulate spindle-mediated memory reprocessing during sleep (Fig 6).

Even during SWS, the brain preserves its capacity to process external sensory information, which is a prerequisite for sensory memory cues to reactivate their associated memories [25]. Cross-species studies have consistently demonstrated that the primary auditory cortex neuronal responses are well preserved during sleep, with little modulation by different vigilance or consciousness states [26,48,49]. Human research further shows that the sleeping brain can process meaningful auditory stimuli [50–53], which are manifested by increased EEG power in both the low-frequency range and the sigma band immediately following auditory stimuli [54–56]. Consistent with these previous studies, we found that both the memory cues and control cues enhanced low-frequency activities (2 to 9 Hz), followed by the extended sigma band activities (11 to 18 Hz) within approximately the first 2 s post-cue onset. While previous studies had reported greater EEG power in response to memory cues than control cues [20,57], we did not find significant differences. This discrepancy might be due to the novelty of the control cues: Whereas in previous studies, control cues were not presented before the TMR session. Here, we presented all memory and control cues at least twice during the pre-learning familiarization phase, in which participants were asked to familiarize the stimuli that would be used in the following associative learning, tests and the sleep TMR session (see Materials and methods). One possibility is that such familiarization of control cues would evoke strong K-complex activity in the sleeping brain [58–60], thus reducing the differences between memory cues and control cues.

Notably, individual differences in early cue-elicited EEG power (within the first 2 s post-cue), regardless of memory cues or control cues, predicted the post-sleep cueing effect. These findings are consistent with previous research that has linked this early cue-elicited EEG power with the post-sleep TMR effects [30,61,62]. However, the early cue-elicited EEG responses were neither different between memory cues and control cues nor between post-sleep remembered and forgotten items within individual participants. Beyond the cue-elicited EEG power, we also found the early cue-elicited EEG contained highly specific, item-level representations. Again, the early item-specific representations were identified regardless of post-sleep memory performance and regardless of memory cues or control cues. These early EEG responses and item-specific representations may thus reflect the effective processing of auditory cues during sleep. It is possible that the early and stronger neural responses to cues would pave the way for subsequent reprocessing of cue-target memories, leading to better memory performance for cued items. Corroborating this hypothesis, we found that across participants, cue-elicited early EEG responses positively predicted the post-sleep memories for untested items.

More importantly, we found compelling evidence that TMR cue-elicited late item-specific representations (2,500 to 2,960 ms post-cue) were associated with post-sleep cue-target memory recall. Specifically, cues of post-sleep remembered items exhibited greater item-specific representations than forgotten items and control cues in a later time window. This difference in item-specific representations cannot be exclusively attributed to different levels of familiarity with cues, given that the cues for post-sleep remembered and forgotten items were repeated the same number of times during pre-sleep learning. In addition, the direct comparisons between the item-specific representations of forgotten items and control items did not yield significant differences. Intriguingly, we found that both early and late item-specific representations were mainly driven by the 0.5 to 4 Hz slow-wave EEG activity post-cue. While mounting evidence has suggested that slow-wave activity plays a crucial role in memory consolidation [63,64], its precise function remains elusive. Our study provides new evidence that slow-wave activity contributed to both the processing of individual auditory cues and the subsequent reprocessing of cue-target memory during sleep. However, it shall be noted that the current study does not allow us to link the cue-elicited neural activity and item-specific representations with the neural activity of word-picture learning during wakefulness. Therefore, it remains an open question whether the memory-relevant late item-specific representations reflect the reactivation of target picture memories or the cue-target pair memories.

TMR cues could induce item-specific representations via a cortical–hippocampal–cortical loop [65–67]: Effective early processing of auditory cues in the neocortex reactivates their associated memory traces that are temporarily stored in the hippocampus [11,68–70]. The reactivated hippocampal memory traces then trigger the reinstatement of the neocortex-dependent memory representations, which may follow the same principle of pattern completion as during wakefulness retrieval [71,72]. This cortical–hippocampal–cortical loop during NREM sleep relies on the timely coupling of thalamocortical spindles to the up-state of SOs in both humans and rodents, serving as the foundation for systems memory consolidation [3,73–76]. Critically, our results showed that item-specific representations during the late window, supported by spindles, predominantly contribute to successful long-term memory. Moreover, the accompanying spindles were preferentially coupled to the up-state of SOs, which may have facilitated cross-region interactions for reactivated memories, particularly the pre-sleep untested items, to be consolidated [77–79]. A more precise delineation of the spindle-SO-coordinated cross-regional interaction awaits future investigations using methods affording both high spatial and temporal resolutions (e.g., intracranial EEG recordings).

Pre-sleep testing modulated the spindle-mediated memory reprocessing during sleep. Given that pre-sleep testing largely boosted memory performance relative to untested items in the current study, it is possible that pre-sleep tested items already underwent retrieval-induced fast consolidation before sleep [32]. Thus, spindles may preferentially support memory reprocessing for untested memories. Corroborating this possibility, previous studies have shown that strong memories did not further benefit from subsequent spontaneous sleep [80] or sleep TMR [36]. Moreover, sleep spindles would preferentially consolidate weak over strong memories [38], and TMR promoted consolidation for memories with low [35] or moderate [36] pre-sleep accuracy. Consistent with this idea, our findings revealed that cue-elicited early EEG power was associated with post-sleep cueing effects only for pre-sleep untested items, which showed an overall weaker memory as compared with tested items. Moreover, increased spindle activity preferentially supported the item-specific representations for these untested items (but not for tested items) during the late time windows associated with memory reprocessing.

Although the observed item-specific representations in the late time window were associated with post-sleep memory, TMR during SWS showed no overall benefit in post-sleep memory tests. A few reasons may explain these results: First, among post-sleep remembered cues, which showed greater item-specific representations than forgotten cues, a substantial portion (65.39%) were from pre-sleep tested items. These items may have undergone retrieval-induced fast consolidation processes before sleep [32], rendering them less likely to further benefit from TMR and sleep [34–36,81]. Supporting this notion, our results indeed indicated that across participants, auditory cue-elicited spectral power predicted post-sleep memory for pre-sleep untested items, but not for pre-sleep tested items. Second, memory consolidation can span days and even months [82]. It is possible that the impact of TMR on memory consolidation, especially for pre-sleep tested items, may not become evident in the immediate post-sleep test [83]. Indeed, recent research showed that TMR cueing benefits emerged 10 days post-encoding but not within the first 12 h [20,84]. Future studies shall consider examining the TMR effect over a longer delay to capture possible long-term benefits. Third, given that TMR cueing effects are highly sensitive to memory measures [10], more precise measurements are desirable to detect the TMR-induced behavioral benefits (e.g., the error distance measures [11,12]). Relatedly, instead of the category report used in the present cued recall test, future word-picture or naturalistic episodic memory paradigms could include verbal recall to examine how TMR impacts perceptual and conceptual details of episodic memories.

In conclusion, the identification of item-specific neural representations and the segregated processing stages contributed to the mechanistic understanding of how exogenous sensory cues trigger memory reprocessing during NREM sleep. Specifically, the early effective processing of individual memory cues may drive subsequent spindle-mediated item-specific representations that contribute to successful long-term memory. These findings may ignite new development of sleep-based memory editing techniques [85] in perturbing individual memories: by targeting the underlying neural activity during the critical memory reprocessing time window, techniques can either strengthen newly acquired knowledge or weaken maladaptive memories.

## Materials and methods

### Ethics statement

All participants gave written informed consent prior to the experiment. The study was approved by the Human Research Ethics Committee of the University of Hong Kong (EA1904004).

### Participants

Thirty healthy, right-handed participants were included in the analyses (23 females, mean ± SD age, 22.37 ± 2.94 years). To obtain reliable results in the EEG power spectral analysis as well as the multivariate RSA for both the memory cues and control cues, we set a minimum requirement of 20 trials. Thus, 7 additional participants who failed to meet this criterion due to insufficient or unstable N2 and N3 sleep were excluded from the analysis. All included participants received multiple rounds of TMR cueing, in the range of [4.96,16]. They had normal or corrected-to-normal vision. Participants were pre-screened on sleep by using the questionnaires, including the Pittsburgh Sleep Quality Index (PQSI) and the Insomnia Severity Index (ISI). They all reported overall good sleep quality and had not taken any medicines to aid sleep in the past month before the experiment. All participants did not suffer from any neurological or psychiatric disorders.

### Stimuli

A total of 96 two-character Chinese verbs and 96 target pictures were used in the experiment. Each verb was randomly paired with a complex visual picture, resulting in 96 cue (word)-target (picture) pairs. The central element of each picture was from one of 4 categories (i.e., animals, electronic devices, plants, and transportation tools), with 24 pictures in each category. For each picture, a highly similar picture was also selected and served as a lure in the old/new recognition task. For each unique picture, the target and lure were randomly assigned among participants. For verbs, visually presented verbs were only used in the familiarization phase, while aurally presented verbs were used throughout the entire experiment. Four additional two-character Chinese verbs were presented during the familiarization phase but were never paired with any pictures. These verbs served as control cues in the TMR. Auditory sounds of the verbs were generated using the Text-To-Speech of iFLYTEK, with an average duration of 631.70 ms (SD: 55.40 ms).

### Procedure

All participants arrived at the sleep lab around 8:30 PM. Participants completed the following tasks in order: a vigilance task, a stimuli familiarization task, a cue-target associative learning task, and a pre-sleep memory test. Participants then proceeded to sleep (12 AM to 8 AM the next morning), wherein TMR was administered during SWS. After approximately 30 min of waking, participants’ vigilance levels were assessed again, followed by the post-sleep memory test. All behavioral tests were conducted using the PsychoPy (version 2020.2.10).

### Psychomotor vigilance task

Participants’ vigilance levels were assessed using the Psychomotor Vigilance Task (PVT), right after participants arrived at the sleep lab and the next day morning. During the vigilance task, a fixation was presented on the center of the screen with a jitter duration in the range of 2 to 10 s. Then, the fixation was replaced by a counter counting incrementally from 0 in 10 ms increments. Participants were instructed to stop the counter by pressing the space bar immediately after they detected the change of fixation to the number. Response time that appeared on the screen would serve as feedback on the performance. This task lasted for 5 min (see S2 Fig).

### Cue word and picture familiarization task

The familiarization task consists of 2 sessions, a cue word-familiarization session, and a picture-familiarization session. In the cue word-familiarization session, each trial started with a 0.3 s fixation, followed by a 0.5 s blank screen. Afterward, a verb was visually presented on the center of the screen for 2 s, accompanied by its verbalization from the speaker. Participants judged whether the spoken verbs were clear and recognizable by pressing a button. All 100 verbs (96 verbs in the word-picture pairs and 4 verbs as control cues) were randomly presented during this stage, with each verb being presented twice. In the picture familiarization session, each trial started with 0.3 s fixation, followed by a 0.5 s blank screen. Afterward, a picture and its label (e.g., for a panda picture, the label would be “panda”) were presented on the screen for 2 s. Participants indicated whether they were familiar with the picture and its name by pressing a button. Each of the 192 pictures (96 targets + 96 lures) was presented twice. For items that participants indicate unfamiliar will be presented for another 2 rounds. At the end of the task, participants indicated that all stimuli were clearly recognizable and familiar.

### Word-picture associative learning task

Participants learned 96 word-picture pairs. Each learning trial consisted of 3 phases: an encoding phase, a maintenance phase, and a vividness rating phase. During the encoding phase, following a 0.3 s fixation and a black screen jittering between 0.9 and 1.5 s, participants viewed a picture presented in the center of the screen for 2 s while hearing the spoken verb from the speaker. Participants were instructed to pay attention to the details of the picture while memorizing the verb-picture associations during the encoding phase. During the maintenance phase, the picture disappeared, and participants were asked to maintain the picture in their minds as vividly as possible for 3 s while hearing the spoken verb again. In the vividness rating phase, participants indicated the vividness of the mental image of the picture on a 1 (not vivid at all) - 4 (very vivid) point Likert scale by pressing one of 4 buttons. The learning task consisted of 3 blocks, with each block containing 32 unique verb-picture pairs that were repeated 3 times. To reduce the recency effect, participants completed an approximately 5-min math task after the learning task.

### Pre-sleep memory test

To understand whether the pre-sleep testing alters the TMR cueing effect, participants were tested on half of the pairs during the pre-sleep test (i.e., 48 pairs, with 12 pairs from one of the 4 picture categories). This test consisted of a cued recall task and a cued recognition task.

In the cued recall task, each trial started with a 0.3 s fixation, followed by a blank screen (0.9 to 1.5 s). The spoken verb was played, prompting participants to report whether they could retrieve the corresponding pictures or not by pressing the “remember” and “forget” keys. This stage was self-paced so that participants had enough time to recall. Immediately following this “remember” or “forget” response, participants were given 2 s to report the category of the picture by pressing one of 4 buttons, with each button indicating one of 4 categories.

To further encourage participants to remember the detailed picture content, instead of the category information, we further ask participants to perform a cued recognition task. In this recognition task, the same half of the pairs were tested. Specifically, each trial started with a fixation (0.3 s) and was followed by a blank screen (0.9 to 1.5 s). Participants next saw the picture presented on the center of the screen while hearing the spoken verb. Participants were asked to indicate if the picture was the same picture paired with the verb during the previous learning task by pressing the “Yes” or “No” button. Participants completed a total of 192 cued recognition trials. These trials consisted of: (1) 48 “old” trials presenting the same learned verb-picture pairs; (2) 48 “critical lure” trials showing old verbs paired with new pictures that highly resemble the corresponding target pictures; (3) 48 “mixed” trials showing mixed verb-picture pairs, by pairing old verbs with old pictures yet from different learned cue-target pairs; (4) 48 “mixed lure” trials showing mixed verb-lure picture pairs, by pairing old verbs with new pictures that highly resemble the target pictures from different cue-target pairs. The order of trial presentations was randomized in the test.

### TMR during slow-wave sleep

To counterbalance the pre-sleep memory performance between cued and uncued items, we thus selected half of the remembered and half of the forgotten pairs (based on the category report performance in the cued recall task) of the tested items as cued items during TMR. In addition, we randomly selected half of the untested items to be cued in the TMR. Picture categories were balanced across cued versus non-cued conditions. Thus, the TMR session contained 48 verbs from pre-sleep learned 96 word (verb)-picture pairs as memory cues, with additional 4 verbs that were not paired with any pictures as control cues. Half of the memory cues were from tested trials, with the remaining half from untested trials. The control cues served as the baseline to examine retrieval-specific neural activity. Therefore, there were 52 unique sound cues played during sleep.

During the nocturnal sleep, white noise was played in the background throughout the night, with an intensity of approximately 45 dB, measured by a sound-level meter placed at the same position where participants laid their heads on the pillow. Experienced experimenters monitored the EEG signals and visually identified signature EEG events characterizing different sleep stages (e.g., spindles, K-complex, SOs). Upon detecting stable SWS, the experimenter would begin the TMR, which occurred approximately 50.04 min (SD: 37.21 min) after the start of the sleep phase. On average, participants were presented with 8.92 (SD: 3.24) rounds of TMR. In each round of TMR, 52 verb cues were randomly presented with an inter-stimuli interval of 5 ± 0.2 s. After each round of cueing, the order of the TMR cues was shuffled and replayed again. Each round was separated by 30 s. The TMR session continued as long as the participants were in SWS in the first 3 to 4 h of the nocturnal sleep. Cueing was stopped immediately when participants showed signs of micro-arousals, awakening, or changed to N1 sleep or rapid eye movement (REM) sleep. Cueing was resumed after the participants returned to stable SWS.

### Post-sleep memory test

Approximately 30 min after awakening and after the PVT test, participants were tested on all 96 pairs. Similar to pre-sleep memory tests, this test included the cued recall and cued recognition tasks. In addition, there was a closed-eye mental retrieval task between these 2 tasks, in which participants were asked to keep their eyes closed and relax while each of the verbs was randomly played via the speaker (ISI = 5 ± 0.2 s). The current study focuses on the TMR-based memory consolidation process. Therefore, the post-sleep closed-eye mental retrieval data will be reported elsewhere.

Although participants completed several memory tests, we consistently chose the category report performance in the cued recall task as the primary memory outcome in both the pre-sleep and post-sleep memory tests, based on the following reasons. First, a meta-analysis of TMR research has suggested that cued recall performance is more sensitive to the TMR impact compared to recognition and subjective reports [10]. Second, following previous memory research that employed the RSA and subsequent memory approach [24,42], the category report in the cued recall task allows us to examine item-specific representations and their relationship with subsequent memories. Third, in the current study, the category report accuracy in both the pre- and post-sleep tests was significantly greater than the chance level (i.e., 0.25) (all *p*s < 0.001). Furthermore, among those correct category report items, the averaged “remember” subjective rate (pre-sleep: 75.2%; post-sleep: 74.0%) and the subsequent memory recognition accuracy (pre-sleep: 77.56%; post-sleep: 76.9%) were high, suggesting that participants indeed remembered the specific item in addition to its associated category.

When categorizing the items into post-sleep remembered and forgotten items based on the post-sleep category report response, we found that for pre-sleep tested items: the range for post-sleep remembered verb-picture pairs: [17, 45] (median = 30) and the range for post-sleep forgotten pairs: [3, 31] (median = 18). For pre-sleep untested items: the range for post-sleep remembered pairs: [7, 25] (median = 16.5) and the range for post-sleep forgotten pairs: [23, 41] (median = 31.5). Notably, when using different combinations of memory measurements (i.e., (1) subjective report and category report; (2) category report and recognition; (3) subjective report and category report and recognition) to define the post-sleep remembered and forgotten items, we obtained highly similar patterns of item-specific representations (S10 Fig).

### EEG recording and preprocessing

We collected the EEG data throughout the experiment except for during the familiarization and PVT tasks. EEG data were recorded using the amplifier from the eego system (ANT neuro, the Netherlands, https://www.ant-neuro.com) with a sampling rate of 500 Hz from 61 channels (waveguard EEG caps) that were mounted in the International 10 to 20 system. Additionally, there were 2 electrodes placed on the left and right mastoids, respectively, and 1 electrode was placed above the left eye for the EOG measurements. Online EEG recordings were referenced to the default reference channel (i.e., CPz). For sleep monitoring, another 2 electrodes were placed on both sides of the chin to measure the EMG using the bipolar reference. EEG data collection was started after the impedance of all electrodes was lower than 20 KΩ.

Sleep EEG data preprocessing was performed using EEGLAB [86] and Fieldtrip toolboxes [87], as well as in-house code that was implemented in MATLAB (MathWorks). EEG data were first notch filtered at 50 ± 2 Hz, and then bandpass filtered between 0.5 and 40 Hz. Then, continuous sleep EEG data were segmented into 15 s epochs, i.e., form −5 s to 10 s relative to the onset of TMR cues. This long epoch was used to eliminate the edge effect in the later time-frequency analysis. Our main interesting time windows for the sleep data are from 0 to 5 s relative to the TMR cue onset. Bad epochs are marked based on visual inspection and rejected from further analysis. Bad channels were marked and interpolated using spherical interpolation in EEGLAB. Afterward, EEG data were re-referenced to the average of the artifact-free data.

### Time-frequency analysis

We performed time-frequency transformation on the preprocessed EEG data, using the complex Morlet wavelets (6 cycles). Spectral power was extracted from the frequency range of 1 to 40 Hz, with a step of 1 Hz and with the time of interest range of [−1 to 5 s] relative to the TMR cue onset. After the time-frequency transformation, the sampling rate of EEG spectral power data is 100 Hz. We normalized the power data within each frequency bin and each channel by first subtracting the mean power in the baseline time windows ([−1 to −0.5 s] relative to the TMR cue onset) and then dividing the same baseline mean power. Finally, we re-segmented the power data into the 5 s epochs (i.e., [0 to 5 s] relative to the TMR cue onset).

### Spindle detection

Individual spindles were detected during the TMR periods following previous studies [29]. Specifically, artifact-free EEG data during the TMR periods were first bandpass filtered between 11 and 16 Hz by using the fourth order two-pass Butterworth filter. Next, the root mean square (RMS) values were calculated for each time point with a moving window of 400 ms. Third, the spindle amplitude criterion is defined as the mean + 1.5 SD of the RMS signal. Sleep spindles were detected if the RMS signal consecutively exceeded the amplitude criterion for a duration of 0.5 to 3 s. Previous studies indicated that spindle activity was prominent over anterior-posterior midlines [88]. To this end, spindle detection was performed for all 7 midline EEG channels (i.e., “FPz”, “Fz”, “FCz”, “Cz”, “Pz”, “POz”, and “Oz”), separately. We then assign the spindle value of 1 to the time points where a spindle was detected and 0 otherwise. The spindle probability was computed as the mean spindle values across trials for each time point in the time range of [0 to 5 s] relative to the TMR cue onset and then averaged across all midline channels.

### Slow-oscillation detection

The detection of SOs was performed on the artifact-free EEG data during the TMR periods. We first bandpass filtered the EEG data between 0.3 and 1.25 Hz using the two-pass FIR filter, with the order equalling the 3 cycles of the low-frequency cut-off. We then detected the zero-crossings in the filtered signal, and the event durations were calculated as the temporal distance between 2 successive positive-to-negative zero-crossings. SO was detected if the peak-to-peak amplitude was greater than the 75th percentile of the absolute amplitude of the filtered signal [89] and the event duration was between 0.8 s and 2 s. SOs detection was performed on the Fz, where the amplitude of SOs was prominent according to previous studies [90].

### SO-spindle coupling

For TMR trials that showed both the spindle and SOs in the time windows (i.e., 2,500 to 2,960 ms), which showed significant interactions of item-specific neural representations and post-sleep memory effect, we then performed the SO-spindle coupling. We first filtered the SO-spindle trials in the SO frequency range (i.e., 0.3 to 1.25 Hz). We then applied the Hilbert transform to the filtered data to extract the instantaneous phase of the SOs. To obtain the amplitude of the spindle activities, we filtered the SO-spindle trials in the spindle frequency range (i.e., 11 to 16 Hz) and then applied the Hilbert transform to the filtered data to obtain the instantaneous amplitude of the spindle activities on an extended time window (i.e., [2.2 to 4.2 s] relatively to TMR cue onset to ensure at least half of the SOs cycle was included). We detected the preferred SO phase, which is concurrent with the maximal spindle amplitude across all trials in each participant, and then tested the distribution of the preferred SO phases across participants against the uniform distribution using the Rayleigh test (CircStat toolbox [91]).

### Representational similarity analysis

We performed RSA between every 2 clean TMR trials that originated from different TMR rounds. To characterize the dynamic change of representational similarity across time, RSA was performed on the preprocessed raw EEG using sliding time windows of 500 ms, with an incremental step of 10 ms, resulting in 250 * 61 (time points * channels) features in each time window. Then, for each time window, we calculated the similarity between vectorized features of every 2 trials that were from different TMR rounds using Spearman’s correlation. All the correlation values were Fisher *Z*-transformed before further statistical analysis.

To examine item-specific representations, we computed the WI similarity and BI similarity. Specifically, WI similarity was calculated as the whole-brain EEG pattern similarity between trial pairs with the same cue, and the BI similarity was calculated as the pattern similarity between trial pairs with different cues. For memory cues, we found 1,859 ± 1,383 WI trial pairs and 87,377 ± 65,002 BI trial pairs. For control cues, we found 170 ± 126 WI trial pairs and 596 ± 454 BI trial pairs. We then averaged the WI similarity and BI similarity across trial pairs within each participant and contrasted the WI similarity versus BI similarity using the paired-sample *t* test across participants for memory and control cues, respectively. Moreover, among memory cues, we found 925 ± 774 WI trial pairs and 2,236 ± 2,218 BI trial pairs for post-sleep remembered items and 935 ± 715 WI trial pairs and 2,304 ± 2,028 BI trial pairs for post-sleep forgotten items. We next contrasted the item-specific representations (i.e., WI-BI similarity) between post-sleep remembered and forgotten items, which allowed us to examine the relationship between item-specific representations and post-sleep memory.

To rule out the potential effects of unbalanced trial pairs in obtaining item-specific representations, we performed 3 control analyses (S4 Fig). In the first analysis, we balanced the WI and BI trial pairs by randomly selecting an equal number of BI trial pairs to match with the number of WI trial pairs (S4A Fig). In the second analysis, we matched the trial pairs of memory items with those of control items by randomly selecting the same number of WI trial pairs and BI trial pairs from the memory items as were present in each of the WI and BI conditions for the control items (S4B Fig). In the third analysis, we matched the trial pair numbers across 4 memory cue conditions (WI remember, BI remember, WI forget, and BI forget) by randomly selecting the same number of trial pairs from these 4 conditions using the smallest number of trial pairs among them (S4C Fig). In addition, to rule out the possibility that any category-level representations may drive the item-specific representations observed in the analyses, we performed the contrast between within-item similarity versus within-category similarity (i.e., similarities between 2 trials with different pictures from the same category), the contrast between within-item similarity versus between-category similarity (i.e., similarities between 2 trials with pictures from different categories), and the contrast between within-category similarity versus between-category similarity (S5 Fig).

### Statistics

For the behavioral data, we conducted a two-way ANOVA with cueing effect (cued versus uncued) and pre-sleep testing (tested versus untested) as repeated measures in analyzing the post-sleep memory performance. We also performed a two-way ANOVA with TMR and time (pre-sleep versus post-sleep) as repeated measures in analyzing the memory performance for tested items. The paired-sample *t* tests were employed to examine the differences between 2 specific experimental conditions (e.g., memory performance between pre-sleep tested items and post-sleep tested items). The relationship between EEG activities during the TMR period and post-sleep memory performance is assessed by the robust linear regression model, which is less likely to be affected by potential outliers [92].

For the EEG data, multiple comparisons across consecutive time windows were corrected using the cluster-based nonparametric statistical tests [93]. Specifically, statistical tests (e.g., *t* test) were performed between conditions (e.g., WI versus BI or tested versus untested) in individual time (or time-frequency) windows. Adjacent time (or time-frequency) windows with statistical values exceeding a threshold (*p* < 0.05) were combined into contiguous clusters. Cluster-level statistics were computed using the sum of the *t* values within a cluster. To test the significance of the time (or time-frequency) cluster, a distribution of cluster-level statistics under the null hypothesis was constructed by randomly permuting condition labels 1,000 times, and the maximum cluster-level statistic in each permutation was extracted. If no significant cluster was found for a permutation, a value of 0 was assigned for that permutation. The nonparametric statistical significance of a cluster was then obtained by calculating the proportion of cluster-level statistics in the distribution under the null hypothesis that exceeded the empirical cluster-level statistics. For an identified cluster, we also performed a two-way ANOVA with pre-sleep testing and subsequent memory as repeated measures and a two-way ANOVA with item-specific neural representations (WI versus BI similarity) and subsequent memory as repeated measures. The statistical significance level for all the analyses is set as *p*-value < 0.05 or corrected *p*-value for clusters (*p*_cluster_) < 0.05.

## Supporting information

S1 Data Excel spreadsheet with individual numerical data organized into separate sheets corresponding to the following figures and figure panels: 2C, 2D, 3C, 3D, 4C, 5D, S1A–S1C, S2A–S2E, S3A–S3F, S6G, S6H, S8A, and S8B. (XLSX)

S1 Fig Auditory cue-elicited EEG power. **(A–C)** Pre-sleep testing (tested vs. untested) by memory (remember vs. forget) two-way repeated measures ANOVA on memory cue-elicited EEG power showed neither significant interaction effects nor significant main effects in either early clusters (i.e., low-frequency and sigma band clusters in Fig 2) or the late cluster (i.e., reduced sigma power cluster) (all *p*s_FWER_ > 0.107, corrected for post hoc comparisons using family-wise error rate, FWER). **(D)** No significant clusters were found when contrasting auditory cue-elicited EEG power between memory cues and control cues (*p*_cluster_ > 0.294). **(E)** After matching the trial number between memory cues and control cues, the difference in cue-elicited EEG power remained nonsignificant (*p*_cluster_ > 0.441). **(F)** Topography plots for the cue-elicited reduced sigma band power in the late cluster (see Fig 2) for memory cues and control cues, respectively. The data underlying this figure can be found in S1 Data.
(TIF)

S2 Fig Behavioral results. **(A)** Category report accuracy during pre- and post-sleep cued-recall tasks. For pre- and post-sleep cued recall performance, regardless of being tested or not before sleep, whether they were cued or not during sleep TMR, the category report accuracies were significantly above chance (i.e., 0.25, all *p*s < 0.001). For pre-sleep tested items, category report accuracy was higher in the post-sleep test than in the pre-sleep test, irrespective of TMR cueing (*t*(29) = 9.85, *p*_FWER_ < 0.001). Moreover, memory accuracy for tested items was significantly higher than that for untested items in the post-sleep test (*t*(29) = 13.67, *p*_FWER_ < 0.001). A repeated measures ANOVA with TMR (cued vs. uncued) and pre-sleep testing (tested vs. untested) as factors on post-sleep category report accuracy revealed neither a significant interaction effect (*F*(1,29) = 0.15, *p* = 0.700) nor a significant main effect of TMR (cued vs. uncued, *F*(1,29) = 0.11, *p* = 0.739). In addition, for pre-sleep tested items, there was no significant TMR (cued vs. uncued) by time (pre-sleep vs. post-sleep) interaction effect (*F*(1,29) = 0.11, *p* = 0.745). **(B)** Subjective remembering during pre- and post-sleep cued-recall tasks. Subjective remembering was quantified by the ratio of trials that participants reported “remember” during the cued-recall test regardless of the following category report accuracy. The results found that subjective remembering for the tested items in the post-sleep test was greater than that in the pre-sleep test, as well as greater than the untested items in the post-sleep test, irrespective of TMR cueing (Both *p*s_FWER_ < 0.001). Further analyses revealed a significant TMR by pre-sleep testing (tested vs. untested) interaction effect in the post-sleep test and a significant TMR (cued vs. uncued) by time (pre- vs. post-sleep) interaction effect for tested items (all *p*s < 0.022). Simple-effects analyses revealed that both interaction effects were driven by greater subjective remembering for cued than uncued items among the tested items during the post-sleep test (*t*(29) = 3.80, *p*_FWER_ = 0.002). **(C)** Recognition accuracy. Recognition accuracy for tested items in the pre-sleep test was significantly greater than that for the untested items in the post-sleep test (*t*(29) = 3.13, *p*_FWER_ = 0.012). However, there was no significant difference in the accuracy between tested items in the pre-sleep test and the post-sleep test, nor between tested and untested items in the post-sleep test (*p*_FWER_ > 0.192). Further analyses revealed no significant TMR by pre-sleep testing interaction effect in the post-sleep test, nor a significant TMR by time interaction effect for tested items (*p*s > 0.350). **(D)** Recognition *d*-prime. The *d*-prime values for tested items in both the pre-sleep and post-sleep tests were significantly higher than untested items (all *p*s_FWER_ < 0.001). In addition, the *d*-prime values for tested items were greater in the pre-sleep test than in the post-sleep test (*p*_FWER_ < 0.001). Further analyses revealed no significant TMR by pre-sleep testing interaction effect in the post-sleep test, nor a significant TMR by time interaction effect for tested items (all *p*s > 0.116). **(E)** No significant difference was found between the reactivation time for the psychomotor vigilance tasks that were performed before encoding on the pre-sleep night and 30 min post-sleep sleep on the next morning (*t*(29) = −1.84, *p* = 0.075). *: *p* < 0.05; **: *p* < 0.01. The data underlying this figure can be found in S1 Data.
(TIF)

S3 Fig Auditory cue-elicited early EEG power change (within the first 2 s post-cue) positively correlated with post-sleep cueing effect for pre-sleep untested items. For panels A and B, we examined the relationship between cue-elicited power and post-sleep cueing effects using robust linear regression analysis. **(A)** Auditory cue-elicited EEG power (regardless of memory cues or control cues) was not significantly associated with the post-sleep cueing effects for pre-sleep tested items. **(B)** Cue-elicited EEG power (regardless of memory cues or control cues) in both the low-frequency range (2–9 Hz) and sigma band (11–18 Hz) was positively associated with the post-sleep cueing effects for pre-sleep untested items. For panels C–F, we investigated how cue-elicited EEG power impacted post-sleep memory performance among either the tested or untested items, for cued and uncued items, via performing robust linear regression analyses. Specifically, in the linear regression model, we employed the cue-elicited EEG power as the predictor, the pre-sleep memory performance as the covariate (as a measure of an individual’s memory ability), i.e., lm = fitlm (data, “post_sleep_memory ~ cue_elicited_power + memory_ability,” “RobustOpts,” “on”). (C) Memory cue-elicited EEG power was not significantly associated with post-sleep memory performance for pre-sleep tested cued or uncued items. (D) Memory cue-elicited EEG power change in the low-frequency range was positively associated with the post-sleep memory performance for pre-sleep untested cued items while negatively associated with the post-sleep memory performance for uncued items. Similar but nonsignificant effects were found in the sigma band. (E) Control cue-elicited EEG power was not significantly associated with the post-sleep memory performance for pre-sleep tested cued or uncued items. (F) Control cue-elicited EEG power change in the sigma band was positively associated with the post-sleep memory performance for pre-sleep untested cued items while negatively associated with the post-sleep memory performance for uncued items, with similar but nonsignificant effects in the low-frequency range. *: *p* < 0.05; **: *p* < 0.01; ***: *p* < 0.001. The data underlying this figure can be found in S1 Data.
(TIF)

S4 Fig Item-specific representations after controlling the trial pair number between conditions. **(A)** After matching the trial pair number between WI similarity and BI similarity conditions, we still identified a significant cluster that showed item-specific representations for memory items (570–1,470 ms, *p*_cluster_ = 0.003). **(B)** After matching the trial pair number of the WI and BI similarity for memory items with the trial pair number of WI and BI similarity for the control items, respectively, we still found a significant cluster that showed item-specific representations for memory items (520–990 ms, *p*_cluster_ = 0.038). An item type (memory vs. control items) by item-specificity (WI vs. BI) repeated measures ANOVA did not reveal any significant interaction effect cluster (*p*_cluster_ > 0.224). The results are highly consistent with that in Fig 3B in the main text. **(C)** Greater item-specific representations for post-sleep remembered items than forgotten items in a late time window (2,500–2,950 ms, *p*_cluster_ = 0.035) after matching the trial pair numbers across 4 memory cue conditions (WI remember, BI remember, WI forget, and BI forget). These results are highly consistent with that in Fig 4A in the main text, indicating that item-specific representations persisted after matching the trial pair number used in the representational similarity analysis. *: *p*_cluster_ < 0.05; **: *p*_cluster_ < 0.01.
(TIF)

S5 Fig Decomposing item-specific representations between within-item (WI) similarity and between-item (BI) similarity. BI similarity consists of within-category (WC) similarity and between-category (BC) similarity. **(A)** A greater WI than WC similarity was identified in a 550–1,160 ms post-cue time window for memory items (*p*_cluster_ = 0.013). **(B)** Post-sleep remembered items showed a greater WI vs. WC effect than forgotten items in a later time window (2,480–2,960 ms post-cue, *p*_cluster_ = 0.031). **(C)** A greater WI than BC similarity was identified in a 560–1,370 ms post-cue time window for memory items (*p*_cluster_ = 0.009). **(D)** Post-sleep remembered items showed a greater WI vs. BC effect than forgotten items in a later time window (2,510–2,950 ms post-cue, *p*_cluster_ = 0.043). **(E, F)** No significant difference was found between WC and BC similarity for memory items or between the WC vs. BC effects for post-sleep remembered and forgotten items (all *p*s_cluster_ > 0.113). These results are highly consistent with Fig 3B and Fig 4A in the main text. *: *p*_cluster_ < 0.05; **: *p*_cluster_ < 0.01.
(TIF)

S6 Fig Contributions of different frequency bands to the memory cue-elicited item-specific representations. **(A–F)** Item-specific representations following memory cues and the contrast of item-specific representations between post-sleep remembered and forgotten items were examined using the EEG data in different frequency ranges, including slow oscillation (0.3–1.25 Hz), slow-wave activity (0.5–4 Hz), theta (4–7 Hz), alpha (8–12 Hz), sigma (11–18 Hz), and gamma bands (25–40 Hz). EEG activity in both the slow-wave and theta frequency ranges exhibited significant item-specific representations for memory items (slow-wave: 580–1,130 ms post-cue, *p*_cluster_ = 0.026; theta: 200–810 ms post-cue, *p*_cluster_ = 0.015, shaded rectangles). In contrast, the other frequency bands did not show significant item-specific representations (all *p*s_cluster_ > 0.114). In addition, only the EEG activity in the slow-wave frequency range showed significantly greater item-specific representations for post-sleep remembered than forgotten items in a later time window (2,520–2,950 ms post-cue, *p*_cluster_ = 0.028, shaded rectangles), while the other frequency bands did not show such effects (all *p*s_cluster_ > 0.078). **(G)** In the early cluster (i.e., 560–1,350 ms post-cue, Fig 3B in the main text), which showed memory cue-elicited item-specific representations (i.e., 0.5–40 Hz, all bands), only EEG activity in the slow-wave frequency range showed significant item-specific representations (*t*(29) = 3.14, *p* = 0.004), with a similar but nonsignificant trend in the slow-oscillation frequency range (*t*(29) = 2.03, *p* = 0.052). In contrast, no significant item-specific representations were found in other frequency ranges (all *p*s > 0.107). **(H)** In the later cluster (i.e., 2,500–2,960 ms post-cue, Fig 4A in the main text), only EEG activity in the slow-wave frequency range exhibited greater item-specific representations for post-sleep remembered items than forgotten items (*t*(29) = 3.75, *p* < 0.001). A similar but nonsignificant trend was observed in the slow-oscillation frequency range (*t*(29) = 1.94, *p* = 0.062). No significant results were found in other frequency ranges (all *p*s > 0.173). *: *p*/*p*_cluster_ < 0.05; **: *p*/*p*_cluster_ < 0.01; ***: *p*/*p*_cluster_ < 0.001. The data underlying this figure can be found in S1 Data.
(TIF)

S7 Fig Item-specific representations were not modulated by pre-sleep testing. To understand whether the pre-sleep testing influences memory reactivations, we performed a three-way repeated measures ANOVA, with pre-sleep testing (tested vs. untested), item-specificity (WI vs. BI), and post-sleep memory (remember vs. forget) as factors. No significant interaction effect was found in any individual time window (*p* > 0.081). Furthermore, a two-way repeated measures ANOVA with pre-sleep testing and item-specific representations as factors for post-sleep remembered items did not reveal any significant interaction effect (*p*_cluster_ > 0.383).
(TIF)

S8 Fig The onset time and duration of the spindles that occurred within the first 2 s following TMR cues were not different either between tested and untested items or between post-sleep remembered and forgotten items. We extracted the spindle onset time and duration for the spindles that occurred within the first 2 s and then performed testing (tested vs. untested) by subsequent memory (remember vs. forget) repeated measures ANOVA on time and duration, respectively. The results found that neither a significant interaction for the spindle onset time (*F*(1, 29) = 0.71, *p* = 0.408, **A**) nor a significant interaction effect for the spindle duration (*F*(1, 29) = 0.83, *p* = 0.371, **B**). The data underlying this figure can be found in S1 Data.
(TIF)

S9 Fig Spindles in an extended late time window (2.2–4.2 s) were coupled to the up-state of slow oscillations. For trials that showed both the spindles and slow oscillations in the late time window (i.e., 2.2–4.2 s), we extracted the preferred phase of the slow oscillation for each spindle in this time window and calculated the mean phase across trials for each participant (CircStat toolbox). The Rayleigh *Z* test was then used to determine if the distribution of phases deviated from a uniform distribution across participants. The results showed that spindle activities for both subsequently remembered and forgotten tested items **(A and B)** as well as for subsequently remembered and forgotten untested items **(C and D)** were all significantly and preferentially coupled to the up-state of SOs (all *ps* < 0.045). *: *p* < 0.05; **: *p* < 0.01.
(TIF)

S10 Fig Item-specific representations in a later time window consistently predicted post-sleep memory when categorizing remembered versus forgotten items across varied memory measurements. **(A)** Remembered items were identified by “remember” responses in the subjective report and correct responses in the category report, while forgotten items were identified by incorrect category reports. Item-specific representations were greater for post-sleep remembered items than forgotten items in a 2,480–2,930 ms post-cue time window (*p*_cluster_ = 0.017, shaded rectangle). **(B)** Remembered items were identified by correct responses in both the category report and recognition tasks, while forgotten items were identified by incorrect category reports. Item-specific representations were greater for post-sleep remembered items than forgotten items in a 2,550–2,920 ms post-cue time window (*p*_cluster_ = 0.049, shaded rectangle). **(C)** Remembered items were identified by “remember” responses in the subjective report and correct responses in the following category report and recognition tasks, while forgotten items were those incorrect in the category report. Item-specific representations were greater for post-sleep remembered items than forgotten items in a 2,480–2,890 ms post-cue time window (*p*_cluster_ = 0.041, shaded rectangle). *: *p*_cluster_ < 0.05.
(TIF)

## Abbreviations

BI: between-item
EEG: electroencephalogram
FWER: family-wise error rate
ISI: Insomnia Severity Index
NREM: non-rapid eye movement
PQSI: Pittsburgh Sleep Quality Index
PVT: Psychomotor Vigilance Task
REM: rapid eye movement
RMS: root mean square
RSA: representational similarity analysis
SO: slow oscillation
SWS: slow-wave sleep
TMR: targeted memory reactivation
WC: within-category
WI: within-item
