# Supplementary figures and images for: Item-specific neural representations during human sleep support long-term memory
Source: PLoS Biol. 2023 Nov 20;21(11):e3002399. doi: 10.1371/journal.pbio.3002399 (PMC10695382; doi:10.1371/journal.pbio.3002399)

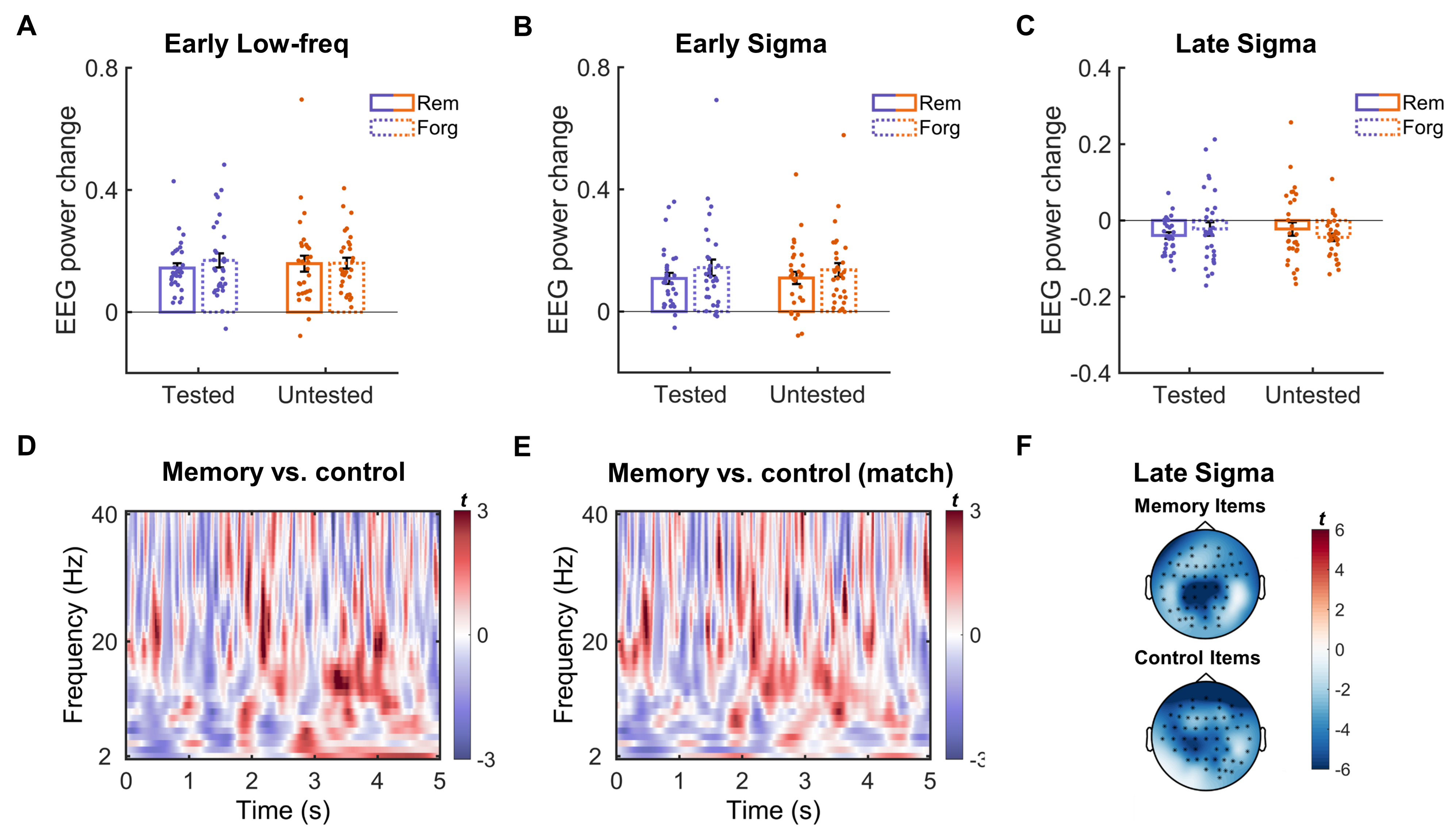

Supplement: S1 Fig — (A–C) Pre-sleep testing (tested vs. untested) by memory (remember vs. forget) two-way repeated measures ANOVA on memory cue-elicited EEG power showed neither significant interaction effects nor significant main effects in either early clusters (i.e., low-frequency and sigma band clusters in Fig 2) or the late cluster (i.e., reduced sigma power cluster) (all psFWER > 0.107, corrected for post hoc comparisons using family-wise error rate, FWER). (D) No significant clusters were found when contrasting auditory cue-elicited EEG power between memory cues and control cues (pcluster > 0.294). (E) After matching the trial number between memory cues and control cues, the difference in cue-elicited EEG power remained nonsignificant (pcluster > 0.441). (F) Topography plots for the cue-elicited reduced sigma band power in the late cluster (see Fig 2) for memory cues and control cues, respectively. The data underlying this figure can be found in S1 Data. (TIF) [file pbio.3002399.s002.tif]

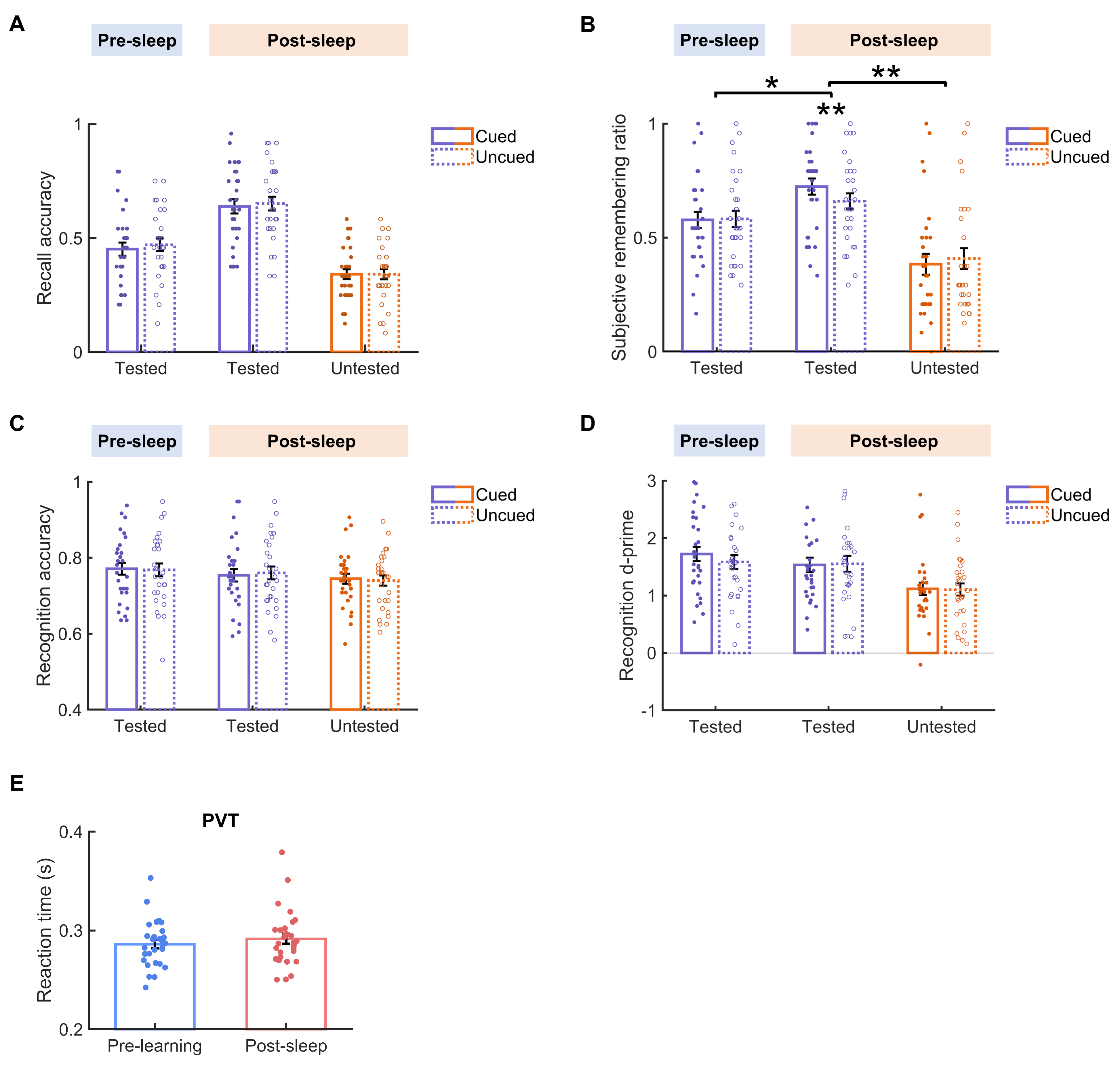

Supplement: S2 Fig — (A) Category report accuracy during pre- and post-sleep cued-recall tasks. For pre- and post-sleep cued recall performance, regardless of being tested or not before sleep, whether they were cued or not during sleep TMR, the category report accuracies were significantly above chance (i.e., 0.25, all ps < 0.001). For pre-sleep tested items, category report accuracy was higher in the post-sleep test than in the pre-sleep test, irrespective of TMR cueing (t(29) = 9.85, pFWER < 0.001). Moreover, memory accuracy for tested items was significantly higher than that for untested items in the post-sleep test (t(29) = 13.67, pFWER < 0.001). A repeated measures ANOVA with TMR (cued vs. uncued) and pre-sleep testing (tested vs. untested) as factors on post-sleep category report accuracy revealed neither a significant interaction effect (F(1,29) = 0.15, p = 0.700) nor a significant main effect of TMR (cued vs. uncued, F(1,29) = 0.11, p = 0.739). In addition, for pre-sleep tested items, there was no significant TMR (cued vs. uncued) by time (pre-sleep vs. post-sleep) interaction effect (F(1,29) = 0.11, p = 0.745). (B) Subjective remembering during pre- and post-sleep cued-recall tasks. Subjective remembering was quantified by the ratio of trials that participants reported “remember” during the cued-recall test regardless of the following category report accuracy. The results found that subjective remembering for the tested items in the post-sleep test was greater than that in the pre-sleep test, as well as greater than the untested items in the post-sleep test, irrespective of TMR cueing (Both psFWER < 0.001). Further analyses revealed a significant TMR by pre-sleep testing (tested vs. untested) interaction effect in the post-sleep test and a significant TMR (cued vs. uncued) by time (pre- vs. post-sleep) interaction effect for tested items (all ps < 0.022). Simple-effects analyses revealed that both interaction effects were driven by greater subjective remembering for cued than u [file pbio.3002399.s003.tif]

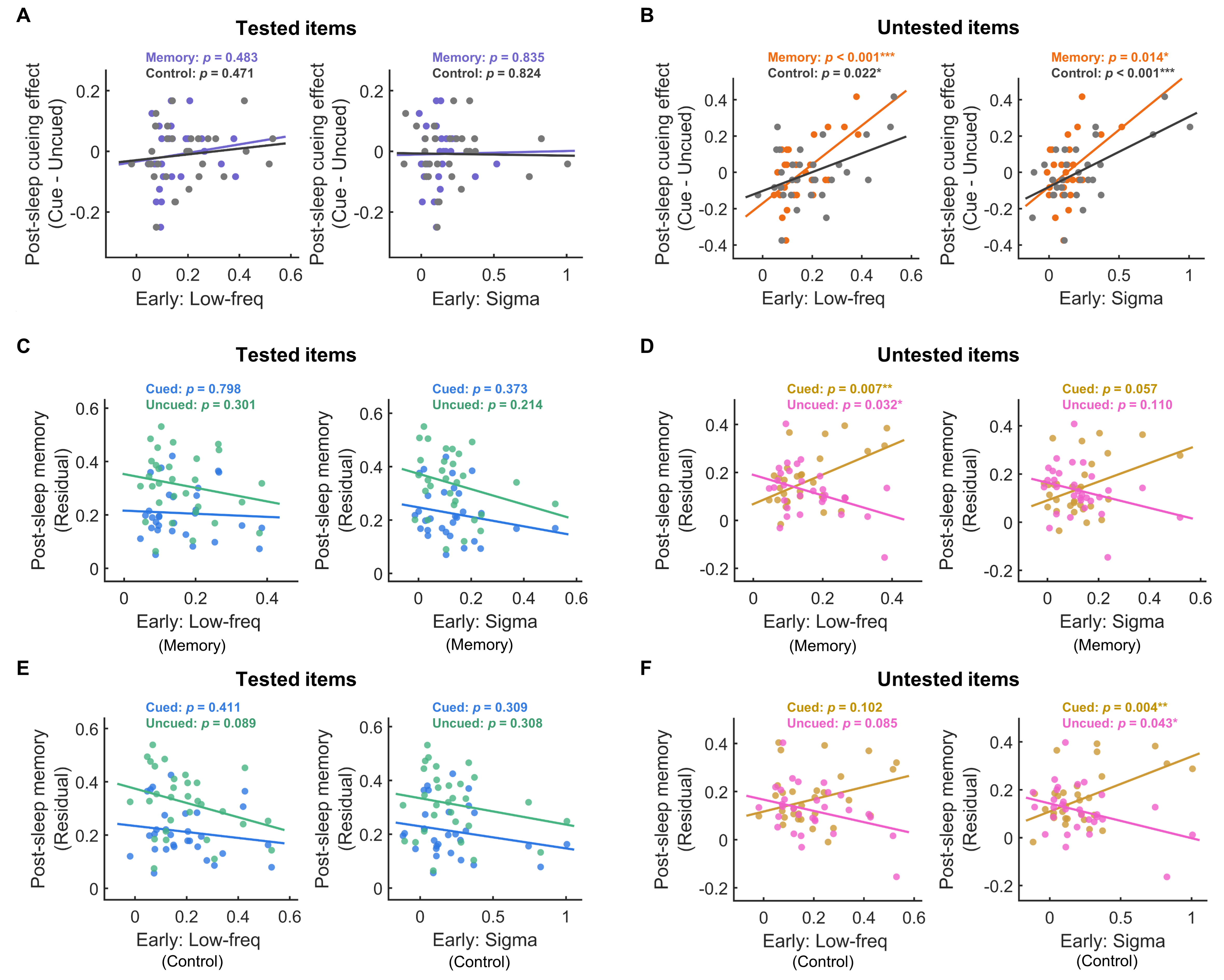

Supplement: S3 Fig — For panels A and B, we examined the relationship between cue-elicited power and post-sleep cueing effects using robust linear regression analysis. (A) Auditory cue-elicited EEG power (regardless of memory cues or control cues) was not significantly associated with the post-sleep cueing effects for pre-sleep tested items. (B) Cue-elicited EEG power (regardless of memory cues or control cues) in both the low-frequency range (2–9 Hz) and sigma band (11–18 Hz) was positively associated with the post-sleep cueing effects for pre-sleep untested items. For panels C–F, we investigated how cue-elicited EEG power impacted post-sleep memory performance among either the tested or untested items, for cued and uncued items, via performing robust linear regression analyses. Specifically, in the linear regression model, we employed the cue-elicited EEG power as the predictor, the pre-sleep memory performance as the covariate (as a measure of an individual’s memory ability), i.e., lm = fitlm (data, “post_sleep_memory ~ cue_elicited_power + memory_ability,” “RobustOpts,” “on”). (C) Memory cue-elicited EEG power was not significantly associated with post-sleep memory performance for pre-sleep tested cued or uncued items. (D) Memory cue-elicited EEG power change in the low-frequency range was positively associated with the post-sleep memory performance for pre-sleep untested cued items while negatively associated with the post-sleep memory performance for uncued items. Similar but nonsignificant effects were found in the sigma band. (E) Control cue-elicited EEG power was not significantly associated with the post-sleep memory performance for pre-sleep tested cued or uncued items. (F) Control cue-elicited EEG power change in the sigma band was positively associated with the post-sleep memory performance for pre-sleep untested cued items while negatively associated with the post-sleep memory performance for uncued items, with similar but nonsignificant effects in the low-frequency range. [file pbio.3002399.s004.tif]

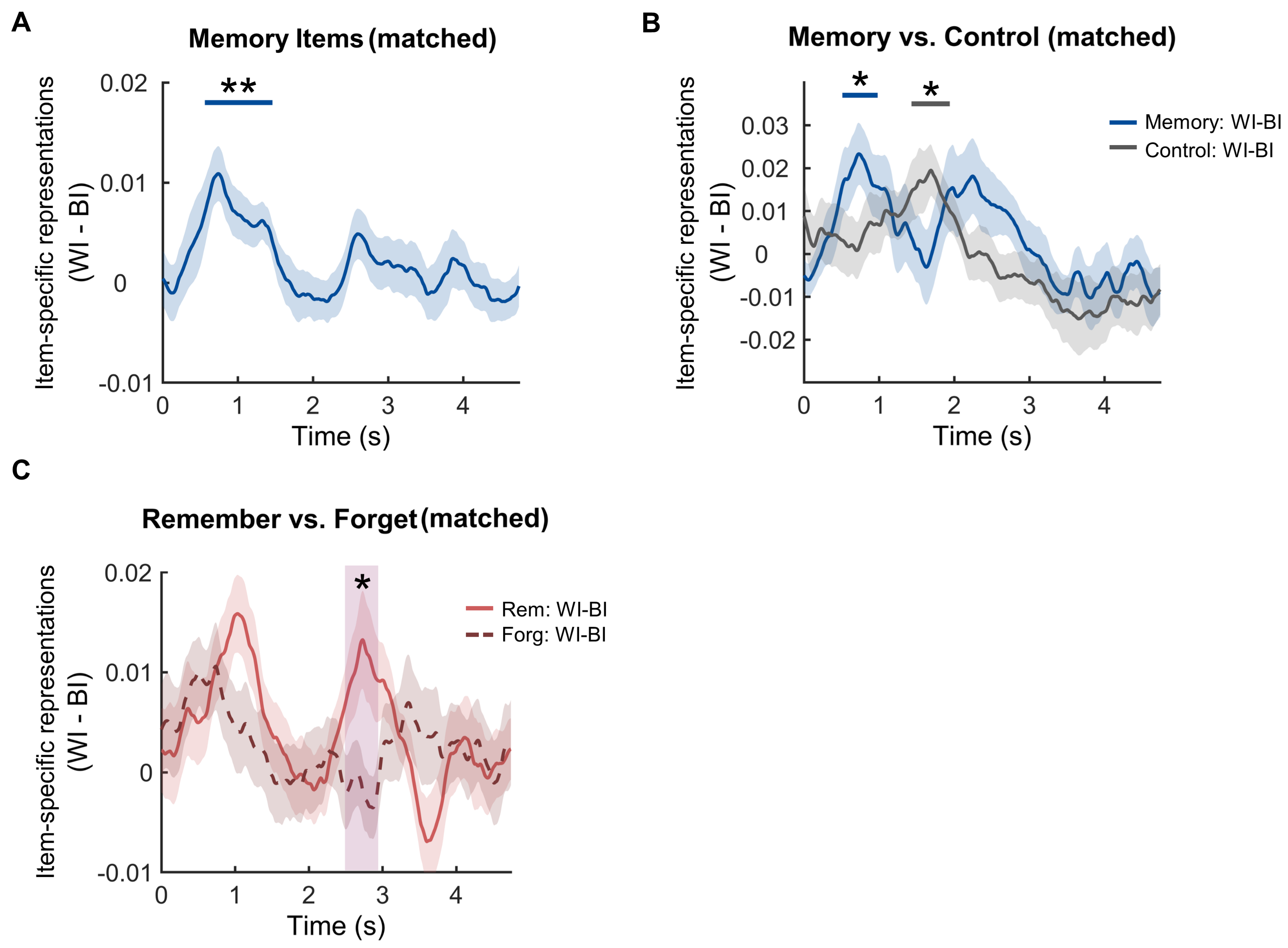

Supplement: S4 Fig — (A) After matching the trial pair number between WI similarity and BI similarity conditions, we still identified a significant cluster that showed item-specific representations for memory items (570–1,470 ms, pcluster = 0.003). (B) After matching the trial pair number of the WI and BI similarity for memory items with the trial pair number of WI and BI similarity for the control items, respectively, we still found a significant cluster that showed item-specific representations for memory items (520–990 ms, pcluster = 0.038). An item type (memory vs. control items) by item-specificity (WI vs. BI) repeated measures ANOVA did not reveal any significant interaction effect cluster (pcluster > 0.224). The results are highly consistent with that in Fig 3B in the main text. (C) Greater item-specific representations for post-sleep remembered items than forgotten items in a late time window (2,500–2,950 ms, pcluster = 0.035) after matching the trial pair numbers across 4 memory cue conditions (WI remember, BI remember, WI forget, and BI forget). These results are highly consistent with that in Fig 4A in the main text, indicating that item-specific representations persisted after matching the trial pair number used in the representational similarity analysis. *: pcluster < 0.05; **: pcluster < 0.01. (TIF) [file pbio.3002399.s005.tif]

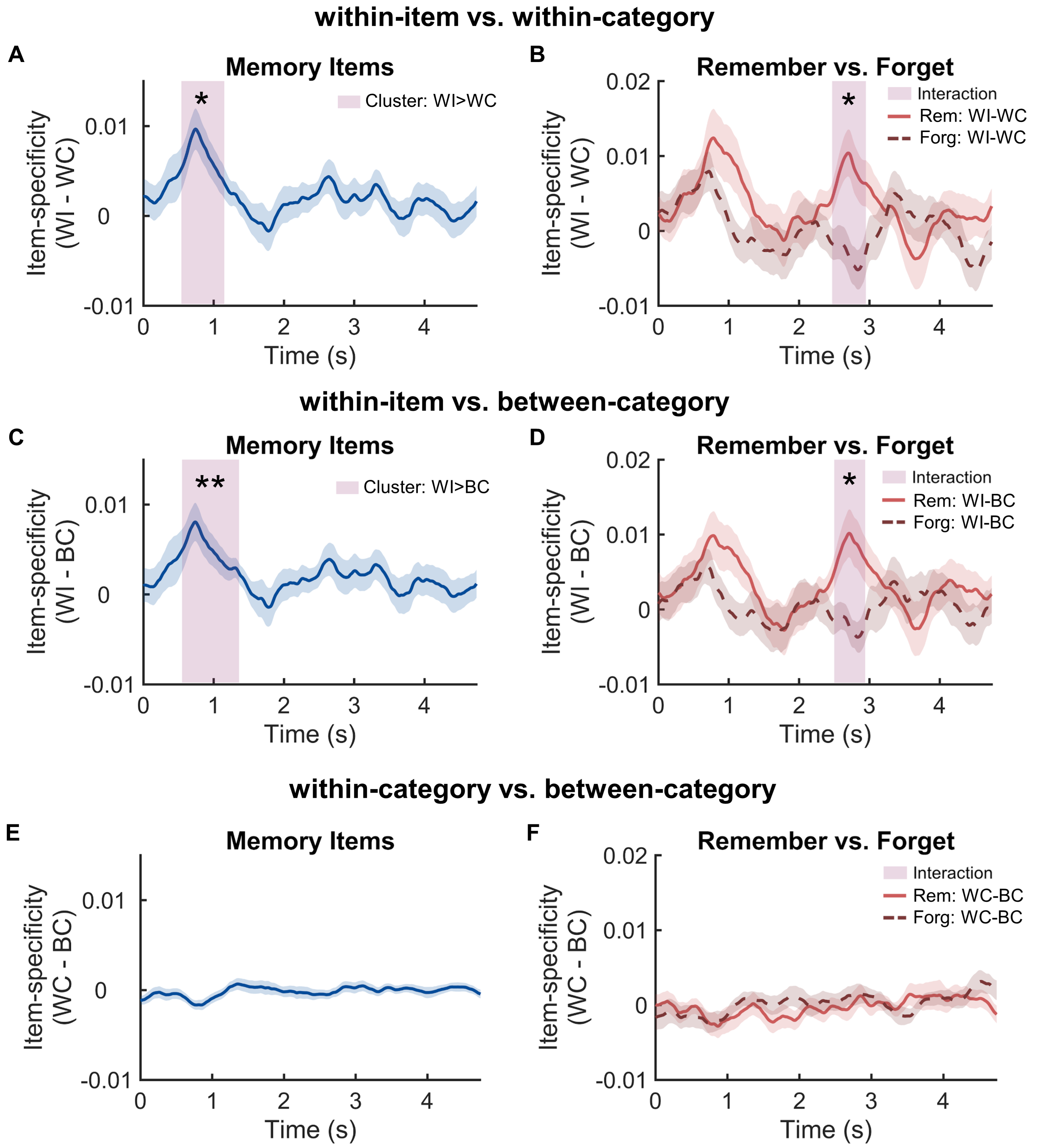

Supplement: S5 Fig — BI similarity consists of within-category (WC) similarity and between-category (BC) similarity. (A) A greater WI than WC similarity was identified in a 550–1,160 ms post-cue time window for memory items (pcluster = 0.013). (B) Post-sleep remembered items showed a greater WI vs. WC effect than forgotten items in a later time window (2,480–2,960 ms post-cue, pcluster = 0.031). (C) A greater WI than BC similarity was identified in a 560–1,370 ms post-cue time window for memory items (pcluster = 0.009). (D) Post-sleep remembered items showed a greater WI vs. BC effect than forgotten items in a later time window (2,510–2,950 ms post-cue, pcluster = 0.043). (E, F) No significant difference was found between WC and BC similarity for memory items or between the WC vs. BC effects for post-sleep remembered and forgotten items (all pscluster > 0.113). These results are highly consistent with Fig 3B and Fig 4A in the main text. *: pcluster < 0.05; **: pcluster < 0.01. (TIF) [file pbio.3002399.s006.tif]

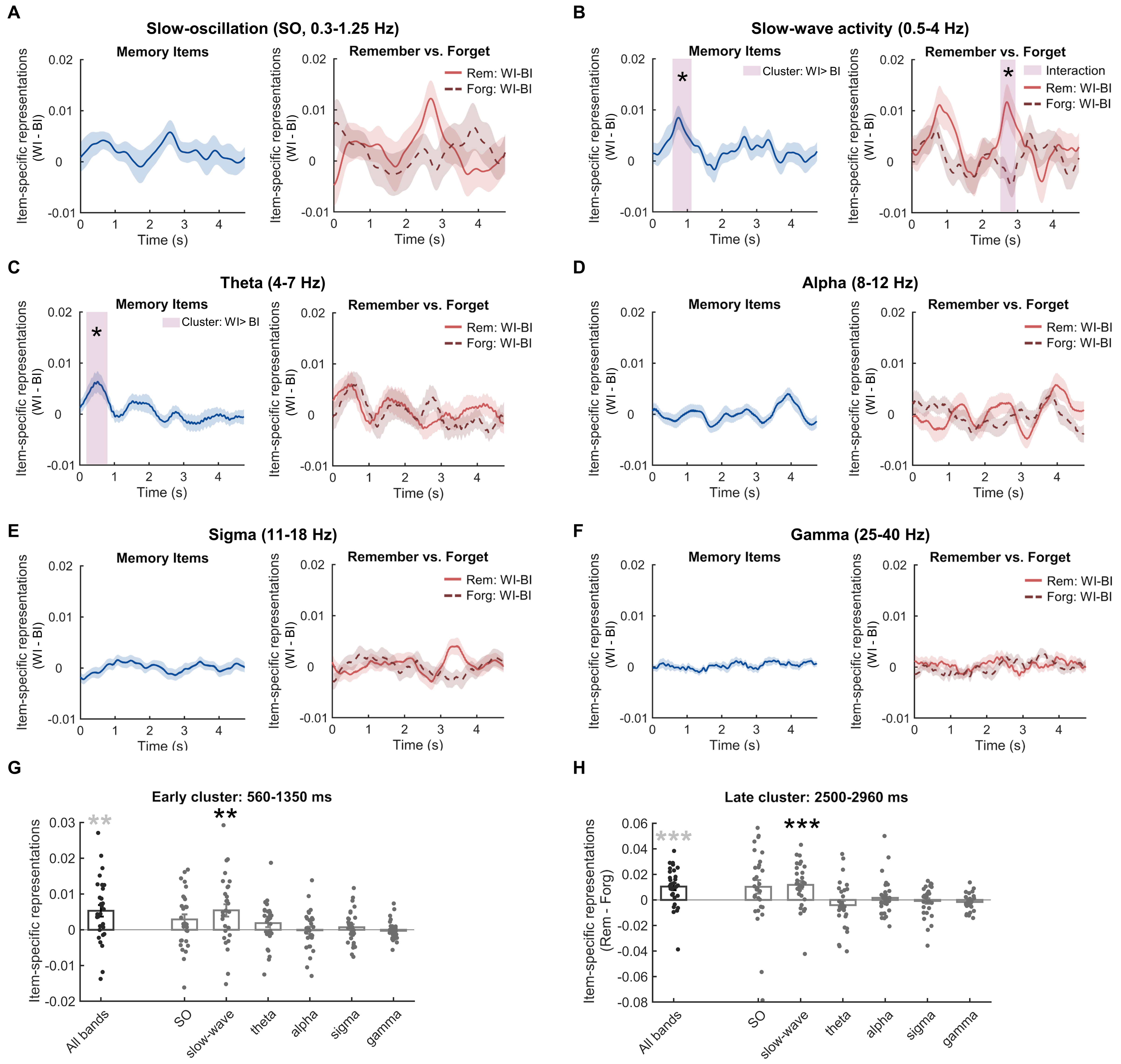

Supplement: S6 Fig — (A–F) Item-specific representations following memory cues and the contrast of item-specific representations between post-sleep remembered and forgotten items were examined using the EEG data in different frequency ranges, including slow oscillation (0.3–1.25 Hz), slow-wave activity (0.5–4 Hz), theta (4–7 Hz), alpha (8–12 Hz), sigma (11–18 Hz), and gamma bands (25–40 Hz). EEG activity in both the slow-wave and theta frequency ranges exhibited significant item-specific representations for memory items (slow-wave: 580–1,130 ms post-cue, pcluster = 0.026; theta: 200–810 ms post-cue, pcluster = 0.015, shaded rectangles). In contrast, the other frequency bands did not show significant item-specific representations (all pscluster > 0.114). In addition, only the EEG activity in the slow-wave frequency range showed significantly greater item-specific representations for post-sleep remembered than forgotten items in a later time window (2,520–2,950 ms post-cue, pcluster = 0.028, shaded rectangles), while the other frequency bands did not show such effects (all pscluster > 0.078). (G) In the early cluster (i.e., 560–1,350 ms post-cue, Fig 3B in the main text), which showed memory cue-elicited item-specific representations (i.e., 0.5–40 Hz, all bands), only EEG activity in the slow-wave frequency range showed significant item-specific representations (t(29) = 3.14, p = 0.004), with a similar but nonsignificant trend in the slow-oscillation frequency range (t(29) = 2.03, p = 0.052). In contrast, no significant item-specific representations were found in other frequency ranges (all ps > 0.107). (H) In the later cluster (i.e., 2,500–2,960 ms post-cue, Fig 4A in the main text), only EEG activity in the slow-wave frequency range exhibited greater item-specific representations for post-sleep remembered items than forgotten items (t(29) = 3.75, p < 0.001). A similar but nonsignificant trend was observed in the slow-oscillation frequency range (t(29) = 1.94, p = 0.062). No significant [file pbio.3002399.s007.tif]

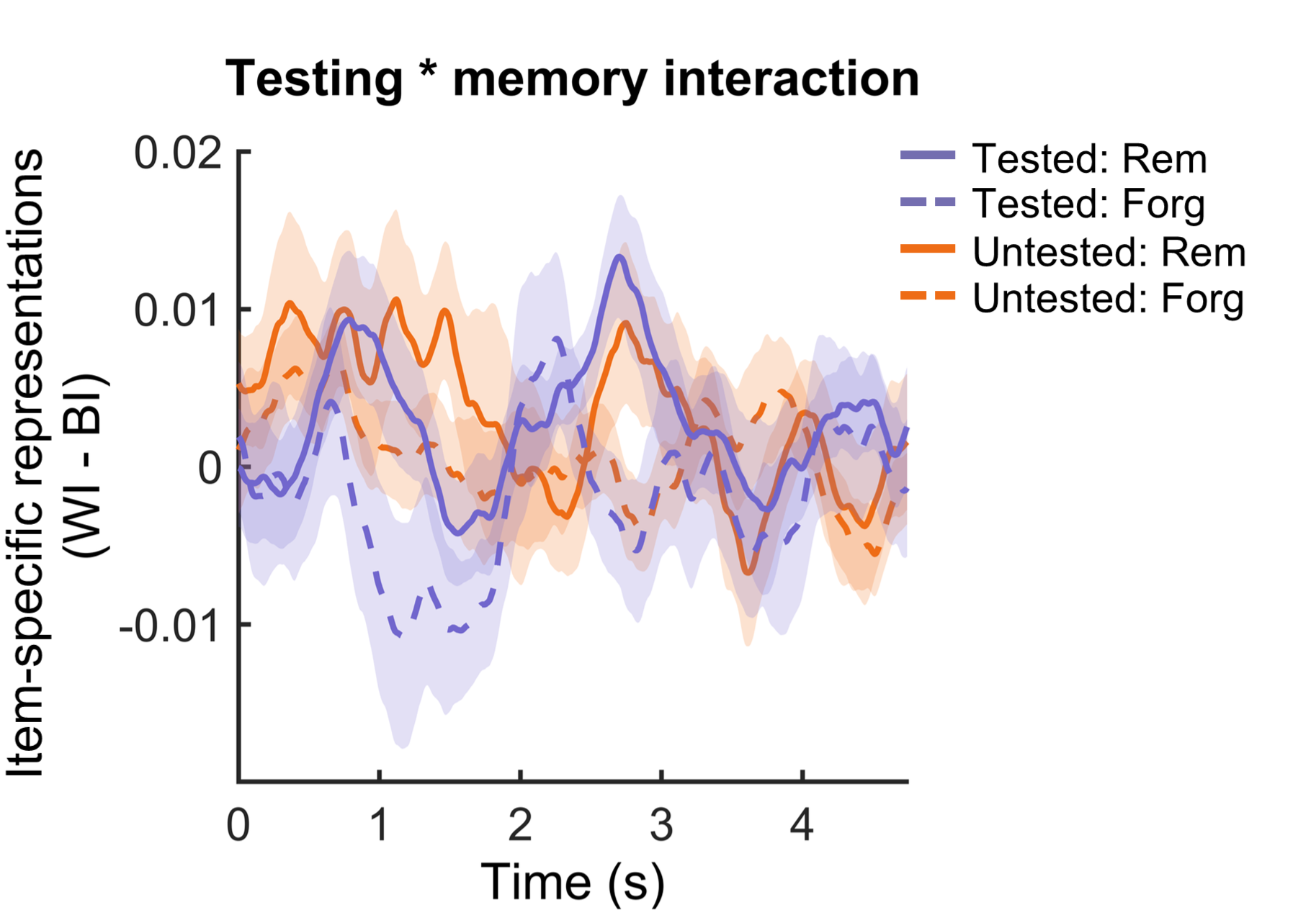

Supplement: S7 Fig — To understand whether the pre-sleep testing influences memory reactivations, we performed a three-way repeated measures ANOVA, with pre-sleep testing (tested vs. untested), item-specificity (WI vs. BI), and post-sleep memory (remember vs. forget) as factors. No significant interaction effect was found in any individual time window (p > 0.081). Furthermore, a two-way repeated measures ANOVA with pre-sleep testing and item-specific representations as factors for post-sleep remembered items did not reveal any significant interaction effect (pcluster > 0.383). (TIF) [file pbio.3002399.s008.tif]

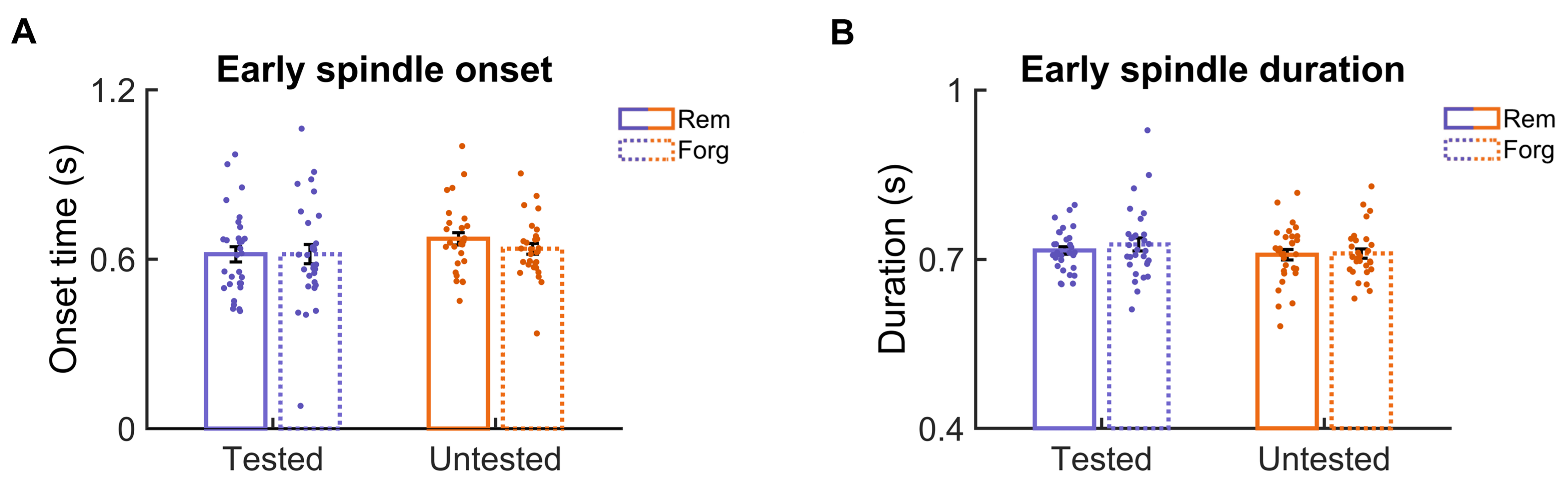

Supplement: S8 Fig — We extracted the spindle onset time and duration for the spindles that occurred within the first 2 s and then performed testing (tested vs. untested) by subsequent memory (remember vs. forget) repeated measures ANOVA on time and duration, respectively. The results found that neither a significant interaction for the spindle onset time (F(1, 29) = 0.71, p = 0.408, A) nor a significant interaction effect for the spindle duration (F(1, 29) = 0.83, p = 0.371, B). The data underlying this figure can be found in S1 Data. (TIF) [file pbio.3002399.s009.tif]

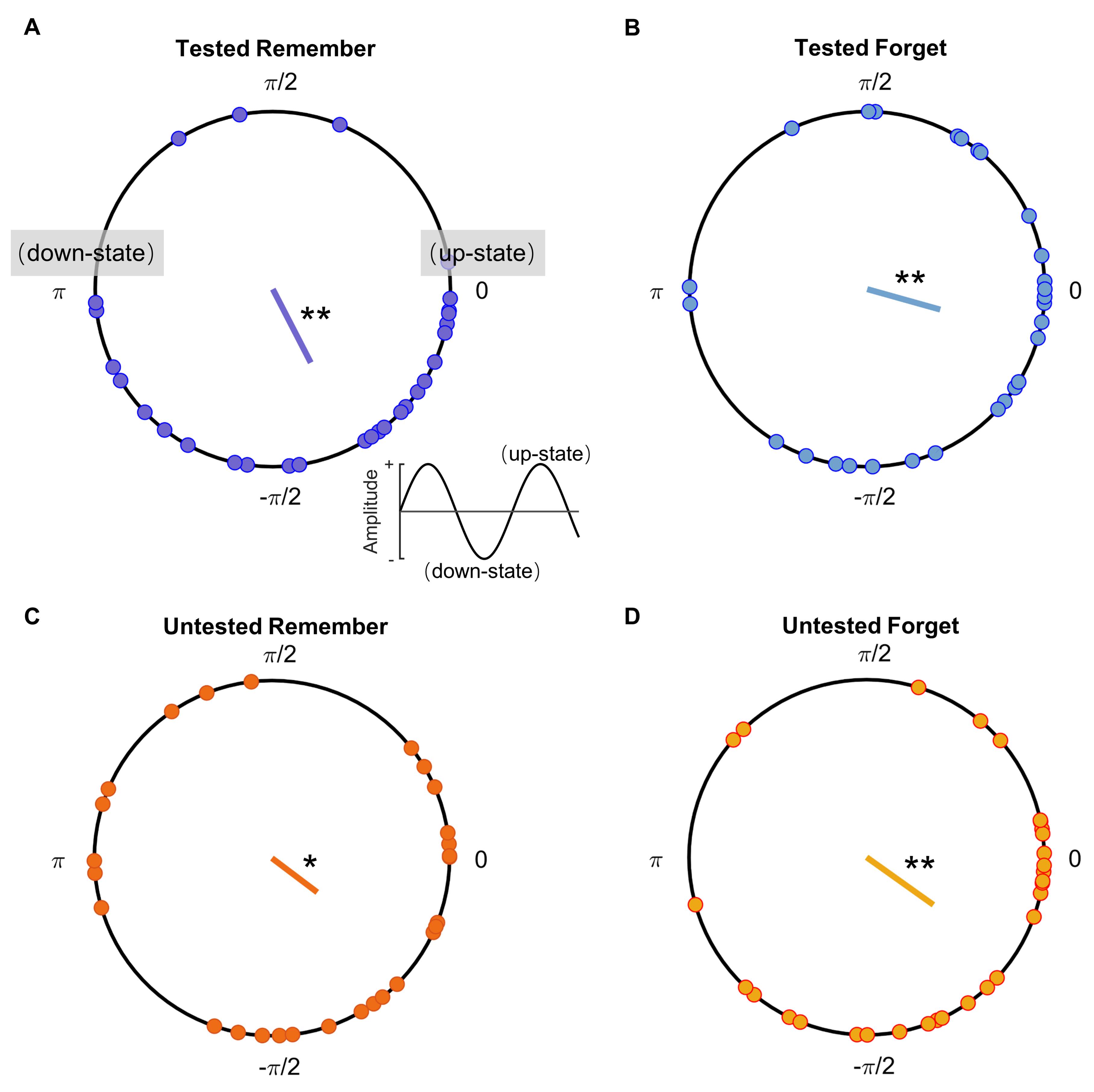

Supplement: S9 Fig — For trials that showed both the spindles and slow oscillations in the late time window (i.e., 2.2–4.2 s), we extracted the preferred phase of the slow oscillation for each spindle in this time window and calculated the mean phase across trials for each participant (CircStat toolbox). The Rayleigh Z test was then used to determine if the distribution of phases deviated from a uniform distribution across participants. The results showed that spindle activities for both subsequently remembered and forgotten tested items (A and B) as well as for subsequently remembered and forgotten untested items (C and D) were all significantly and preferentially coupled to the up-state of SOs (all ps < 0.045). *: p < 0.05; **: p < 0.01. (TIF) [file pbio.3002399.s010.tif]

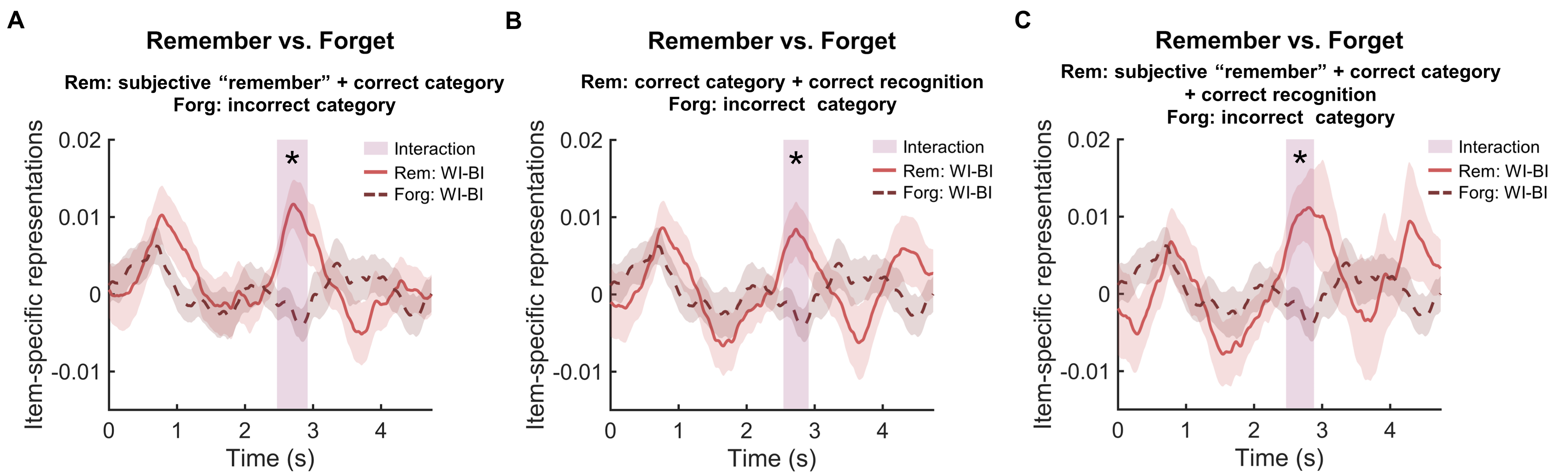

Supplement: S10 Fig — (A) Remembered items were identified by “remember” responses in the subjective report and correct responses in the category report, while forgotten items were identified by incorrect category reports. Item-specific representations were greater for post-sleep remembered items than forgotten items in a 2,480–2,930 ms post-cue time window (pcluster = 0.017, shaded rectangle). (B) Remembered items were identified by correct responses in both the category report and recognition tasks, while forgotten items were identified by incorrect category reports. Item-specific representations were greater for post-sleep remembered items than forgotten items in a 2,550–2,920 ms post-cue time window (pcluster = 0.049, shaded rectangle). (C) Remembered items were identified by “remember” responses in the subjective report and correct responses in the following category report and recognition tasks, while forgotten items were those incorrect in the category report. Item-specific representations were greater for post-sleep remembered items than forgotten items in a 2,480–2,890 ms post-cue time window (pcluster = 0.041, shaded rectangle). *: pcluster < 0.05. (TIF) [file pbio.3002399.s011.tif]
